# Nanocatalytic bacteria disintegration reverses immunosuppression of colorectal cancer

**DOI:** 10.1093/nsr/nwac169

**Published:** 2022-08-29

**Authors:** Han Jiang, Yuedong Guo, Zhiguo Yu, Ping Hu, Jianlin Shi

**Affiliations:** State Key Laboratory of High Performance Ceramics and Superfine Microstructures, Shanghai Institute of Ceramics, Chinese Academy of Sciences; Research Unit of Nanocatalytic Medicine in Specific Therapy for Serious Disease, Chinese Academy of Medical Sciences, Shanghai 200050, China; Center of Materials Science and Optoelectronics Engineering, University of Chinese Academy of Sciences, Beijing 100049, China; State Key Laboratory of High Performance Ceramics and Superfine Microstructures, Shanghai Institute of Ceramics, Chinese Academy of Sciences; Research Unit of Nanocatalytic Medicine in Specific Therapy for Serious Disease, Chinese Academy of Medical Sciences, Shanghai 200050, China; Center of Materials Science and Optoelectronics Engineering, University of Chinese Academy of Sciences, Beijing 100049, China; State Key Laboratory of High Performance Ceramics and Superfine Microstructures, Shanghai Institute of Ceramics, Chinese Academy of Sciences; Research Unit of Nanocatalytic Medicine in Specific Therapy for Serious Disease, Chinese Academy of Medical Sciences, Shanghai 200050, China; Center of Materials Science and Optoelectronics Engineering, University of Chinese Academy of Sciences, Beijing 100049, China; State Key Laboratory of High Performance Ceramics and Superfine Microstructures, Shanghai Institute of Ceramics, Chinese Academy of Sciences; Research Unit of Nanocatalytic Medicine in Specific Therapy for Serious Disease, Chinese Academy of Medical Sciences, Shanghai 200050, China; Shanghai Tenth People's Hospital, Shanghai Frontiers Science Center of Nanocatalytic Medicine, School of Medicine, Tongji University, Shanghai 200092, China; State Key Laboratory of High Performance Ceramics and Superfine Microstructures, Shanghai Institute of Ceramics, Chinese Academy of Sciences; Research Unit of Nanocatalytic Medicine in Specific Therapy for Serious Disease, Chinese Academy of Medical Sciences, Shanghai 200050, China; Center of Materials Science and Optoelectronics Engineering, University of Chinese Academy of Sciences, Beijing 100049, China; Shanghai Tenth People's Hospital, Shanghai Frontiers Science Center of Nanocatalytic Medicine, School of Medicine, Tongji University, Shanghai 200092, China

**Keywords:** tumor-associated bacteria, nanocatalytic bacteria disintegration, immunosuppressive reversal, colorectal cancer immunotherapy

## Abstract

Tumor-associated bacteria (TAB) play a critically important role in regulating the microenvironment of a tumor, which consequently greatly deteriorates the therapeutic effects by chemo- and radiotherapy deactivation and, more considerably, leads to substantial immunosuppression. On the contrary, herein we propose a nanocatalytic tumor-immunotherapeutic modality based on the bacteria disintegration by bacteria-specific oxidative damage under magnetic hyperthermia for highly effective immune response activation-promoted tumor regression. A monodispersed and superparamagnetic nanocatalytic medicine modified by arginyl-glycyl-aspartic acid (RGD) and (3-carboxypropyl)triphenylphosphonium bromide (TPP), named as MNP-RGD-TPP herein, has been synthesized, which features selective accumulation at the TAB by the electrostatic affinity, enabling effective TAB disintegration by the nanocatalytic Fenton reaction producing abundant cytotoxic hydroxyl radicals *in situ* under alternating magnetic field-induced hyperthermia. More importantly, the lipopolysaccharide has been metabolically secreted from the destructed TAB as pathogen-associated molecular patterns (PAMPs) to M1-polarize tumor-associated macrophages (TAMs) and promote the maturation of dendritic cells (DCs) for innate immuno-response activation of TAMs, followed by cytotoxic T lymphocytes awakening under the PAMPs presentation by the mature DCs against tumor cells. The integrated innate and adaptive immunity activations based on this TAB-promoted nanocatalytic immunomedicine, instead of magnetic heating-induced hyperthermia or the released Fe^2+^/Fe^3+^ Fenton agent, has been found to achieve excellent therapeutic efficacy in an orthotopic colorectal cancer model, demonstrating the great potential of such an integrated immunity strategy in clinical tumor immunotherapy.

## Teaser

A nanocatalytic tumor-immunotherapeutic modality based on the bacteria-specific oxidative damage under magnetic hyperthermia (MH) reverses immunosuppression of tumor microenvironment and achieve excellent therapeutic efficacy in an orthotopic colorectal cancer model.

## INTRODUCTION

Ever-increasing amounts of emerging evidence support the fundamental role of microbiome in the development and therapy of cancer [1]. Noticeably, bacteria in the tumor microenvironment (TME) play a non-neglectable role in promoting the development and metastasis of cancer, including laryngeal, esophageal, gastric, colorectal cancer (CRC) and primary liver cancer as well [2]. Despite substantial advances in cancer treatments, less attention has been paid to tumor-associated bacteria (TAB) in the TME that are capable of deteriorating the efficacy of traditional treatments via contributing to resistance and insensitivity to anticancer treatments, such as chemotherapy and radiotherapy [3,4]. Moreover, TAB are also responsible for reinforcing the immunosuppressive effect to help tumor cells escape the attack of the immune system in the TME [5] by e.g. exhausting cytotoxic T lymphocytes and promoting tumor-promoting M2-polarized macrophage infiltration. Evidence indicates that TAB are able to negatively modulate the tumor-immune microenvironment mainly by upregulating the expression of transforming growth factor beta 1 (TGF-β1) and high mobility group box 1 (HMGB1) proteins in tumor cells [6]. In addition, abnormal microbiome composition in the TME can also cause primary resistance to immune checkpoint inhibitors, and distinct and abundant TAB are able to drive suppressive monocytic cellular differentiation in pancreatic cancer leading to T-cell anergy [7]. Notably, nanomedicines provide great opportunities to facilitate cancer immunotherapies for clinical translation in a safe and effective manner [8,9]. Numbers of studies have extensively explored bacteria-based cancer theranostics, including CRC therapy [10–14]. Hence, targeting the microbiome based on nanomedicines may open up a promising and effective route in preventing oncogenesis, reversing intratumoral immunosuppression and immune-sensitizing tumors, which may emerge as a novel strategy of tumor immunotherapy.

The impact of TAB in CRC on immunosuppression is especially prominent in comparison to other types of cancers because of the abundant bacteria environment around the CRC. There has been strong evidence that the crosstalk between homeostatic microbiota and the immune system at the gut level helps to shape the immune system as a whole. It has also been reported that CRC features a certain degree of imbalance in the gut–microbiome axis and is infiltrated by abundant bacteria [15]. Previous studies have identified that *Fusobacterium nucleatum* (*Fn*), the most prevalent bacterial species in CRC, can adhere to the surface of tumor cells by specifically targeting the inhibitory receptor TIGIT, to inhibit immune cell activities [16]. Additionally, dysregulated *Fn* in CRC would drive adaptive immunity dysfunction, leading to immunotherapy resistance and resultant fast tumor progression via inherent disruption of group 3 innate lymphoid cells (ILC3s) [17].

Nevertheless, despite all these detrimental effects of microbiome on tumor development, a certain kind of species on/in the bacteria may help activate the immune system against tumors. As a typical example, lipopolysaccharide (LPS), an important component on the outer wall of gut gram-negative bacteria (G–), is known for its role as a common immune-stimulatory agonist. As typical pathogen-associated molecular patterns (PAMPs), exogenous LPS can be recognized by host pattern-recognition receptors (PRRs), resulting in the promoted local M1 polarization of macrophages and maturation of dendritic cells (DCs) through initiating the Toll-like receptor 4 (TLR4), adaptor myeloid differentiation primary response protein 88 (MyD88) and nuclear factor-κB (NF-κB) pathways [18]. Such a M1 polarization of macrophages and maturation of dendritic cells (DCs) trigger the secretions of abundant proinflammatory cytokines and type I interferon (IFN) to protect tissues from various diseases [19]. Simultaneously, mature DCs will travel to tumor-draining lymph nodes where they interact with and stimulate naive T cells to form T effector cells [20]. Therefore, these peculiarities make the immunogenic LPS of TAB a potential target to reactivate the innate and adaptive immunity against the development of CRC.

**Scheme 1.**
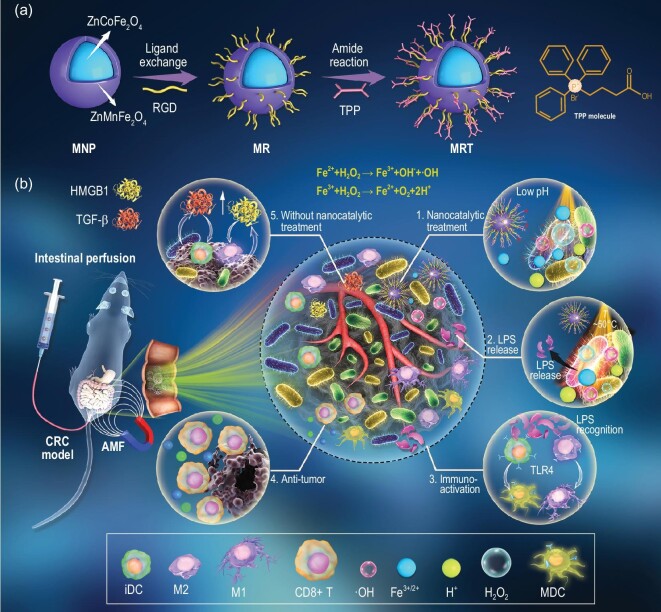
Schematics of MRT synthesis and nanocatalytic antitumor immune activation for colorectal cancer therapy. (a) Synthesis of ZnCoFe_2_O_4_@ZnMnFe_2_O_4_ magnetic nanoparticles (MNP) and further RGD/TPP modification for the synthesis of MRT nanoparticles. (b) The proposed immunotherapeutic strategy based on the nanocatalytic bacteria-promoted immunity: (1–4) The nanocatalytic treatment by MRT+MH leads to the oxidative damage of TAB due to the Fenton reaction and the subsequent LPS fragment release, which acts as a type of PAMPs to reactivate the immune response to achieve the tumor-immunotherapeutic effect. (5) Without the nanocatalytic treatment, TAB actually plays an immunosuppression-promoting role in tumor development by the upregulation of HMGB1 and TGF-β.

In this study, we propose a novel bacteria-stimulated tumor-immunotherapeutic modality by using nanocatalytic magnetic nanoparticles targeting bacterial biofilm, which can activate integrated immunity by bacteria-specific oxidative damage. First, core–shell-structured ZnCoFe_2_O_4_@ZnMnFe_2_O_4_ magnetic nanoparticles (MNP) were synthesized and modified by RGD/TPP ligands (MRT) (Scheme 1a), which feature an ultra-strong magnetism and outstanding magnetic hyperthermia (MH) performance [21]. The obtained MRT nanoparticles exhibit excellent superparamagnetic property at room temperature and can be stably dispersed in aqueous solutions after RGD modification, which is crucial for biomedical applications. Importantly, the designed MRT nanoplatform can specifically target the TAB clustered around tumor cells owing to the electrostatic interactions between MRT and bacteria, and sustainably release the Fe^2+^/Fe^3+^ for potential Fenton reaction under magnetothermal and acidic conditions. As shown in Scheme 1b, the intestinal-perfused MRT can be delivered directly to the tumor site and preferentially adhere onto the negatively charged surface of the bacteria in the TME via the affinity of the lipophilic cationic TPP ligand. Under an alternating magnetic field (AMF), MRT on the surface of the bacterial wall will generate a controlled amount of heat, inducing the vulnerability of dense biofilms to external stimulation [22]. Under the combined effects of magnetic heating and weak acid conditions in the TME, Fenton reagent Fe^2+^/Fe^3+^ released from MRT will react with over-expressed H_2_O_2_ in the TME to generate abundant hydroxyl radicals (•OH) *in situ* on the surface of bacteria, resulting in the disassembly of bacterial biofilms to release LPS. The bacterial LPS will be recognized as an immunogenic PAMPs by PRRs to promote the M1 polarization of TAMs and induce the maturation of immature DCs (iDCs) via the TLR4–MyD88–NF-κB pathway, finally activating T effector cells and triggering the integrated immunity to kill tumor cells. In this work, tumor immunotherapeutics based on bacteria-mediated and nanocatalysis-activated immune responses, rather than the MRT with MH (MRT+MH)-induced hyperthermia or the released Fe^2+^/Fe^3+^ Fenton agent, has been identified to be responsible for the resultant excellent therapeutic efficacy in a CRC model, which sheds light on the significance of nanocatalytic immune activation in cancer immunotherapy and provides a basis for exploring more potent vaccines and adjuvants for antitumor immunity.

## RESULTS

### Synthesis and characterizations of MRT nanoparticles

**Figure 1.**
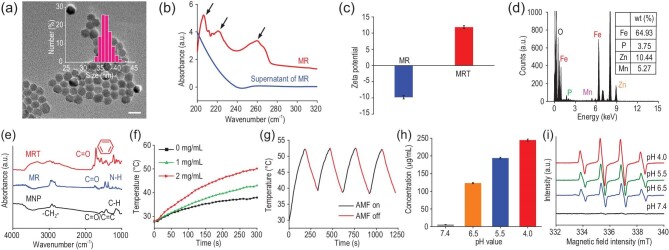
Characterizations of MRT nanoparticles. (a) TEM image of MRT nanoparticles; inset: DLS size distribution in ethanol. Scale bar, 20 nm. (b) The characteristic UV–vis spectra of RGD peptide on the surface of MR dispersed in deionized water before and after centrifugation. (c) Zeta potentials of MR and MRT nanoparticles in an aqueous phase (*n* = 3). (d) Element (O, Fe, Mn, Zn and P) analyses of MRT nanoparticles by EDS; inset: quantitative data of Fe, Mn, Zn and P elements in MRT by ICP-OES. (e) Fourier transform infrared (FT–IR) spectra of oleic acid-coated MNP, RGD-modified MR nanoparticles and further TPP-modified MRT nanoparticles. (f) Temperature–time curves by magnetic heating at different concentrations of MRT in PBS under AMF (1.35 kAm^–1^). (g) The heating curve of MRT in an aqueous phase (4 mg/mL) for four cycles under AMF (1.35 kAm^–1^). (h) The pH-dependent Fe release amounts from MRT after MH in simulated body fluid (SBF) at different pH values. Data are expressed as means ± SD (*n* = 3). (i) ESR spectra evaluating •OH generations in SBF, in which supernatants of MRT nanoparticles after MH at pH = 4.0, 5.5, 6.5 and 7.4 were added in the H_2_O_2_ solution.

The core–shell-structured and Zn^2+^-doped ZnCoFe_2_O_4_@ZnMnFe_2_O_4_ superparamagnetic nanoparticles were synthesized using a seed-mediated method [23] and then functionalized with RGD/TPP molecules to obtain MRT. The transmission electron microscopy (TEM) images in Fig. 1a show uniform quasi-spherical morphology and excellent monodispersity of MRT in aqueous solutions, which is crucial for biomedical applications. Correspondingly, the size distribution of MRT was determined using dynamic light scattering (DLS) measurement, which indicated an average hydrodynamic diameter of ∼37.8 nm (Fig. 1a, inset). During the synthesis of MRT nanoparticles, the monodispersity and morphology of both MNP and MNP-RGD (MR) have been majorly retained (Supplementary Fig. S1a and b). The characteristic polypeptide absorption peaks (215, 220, 256 nm) in the UV–vis spectra of MR demonstrate the successful modification of RGD on the MNP surface (Fig. 1b). Moreover, the oleic acid-coated MNP can be visibly transferred into the aqueous phase from the oil phase after RGD modification, enabling subsequent biomedical application (Supplementary Fig. S1c). Next, TPP molecules were further grafted onto MR nanoparticles to form MRT by an amide reaction, which led to increased zeta potential and the shift of the characteristic absorption of RGD (200–290 nm) in the UV–vis spectra (Fig. 1c and Supplementary Fig. S1d). Moreover, the corresponding energy dispersive spectroscopy (EDS) spectrum of MRT shows the characteristic peak of the P element from the TPP group (Fig. 1d). Quantitative analyses of Fe, Mn, Zn and P elements in MRT nanoparticles were further conducted using inductively coupled plasma optical emission spectrometer (ICP-OES) measurements. The resultant mass percentages of Fe, Mn, Zn and P have been calculated to be 64.93%, 5.27%, 10.44% and 3.75%, respectively (Fig. 1d, inset). Fourier transform infrared (FT–IR) spectroscopy spectrum of MRT show the disappearance of C=C groups (1673 cm^–1^) of oleic acid molecules along with the appearance of an amide bond –CO–NH– (1734 cm^–1^) and benzene ring (1450–1650 cm^–1^) (Fig. 1e), further indicating successful RGD/TPP modifications.

Then, the MH performance of MRT nanoparticles was evaluated at 1.35 kAm^–1^ AMF, which is considered to be biosafe as previously reported [24]. Figure 1f shows the rapidly intensified MH effect of MRT in phosphate-buffered saline (PBS) at increased concentrations. Remarkably, the temperature of the MRT-containing solution at 2 mg/mL increases from room temperature to ∼50°C in 300 s of exposure to AMF. Subsequently, we explored the magnetothermal stability and reversibility of MRT nanoparticles for MH by switching on/off AMF. Figure 1g and Supplementary Fig. S1e show no apparent MH efficacy decay during four AMF on/off cycles, demonstrating the excellent stability and controllability of the MH effect induced by MRT nanoparticles. Additionally, the physical property measurement system was applied to quantify the field-dependent magnetization of MNP/MR/MRT at room temperature. The saturation magnetizations of MNP/MR/MRT are 62.5, 67.6 and 70.2 emu/g, respectively (Supplementary Fig. S1f), indicating substantial magnetization enhancements compared with conventional Fe_3_O_4_ or γ-Fe_2_O_3_ nanoparticles [25]. Furthermore, the hysteresis loops of MNP/MR/MRT nanoparticles show near-zero coercive force under the absence of an external magnetic field, which implies their superparamagnetic property favorable for biomedical applications. The rapid magnetic collection of MRT nanoparticles by a magnet in 60 s also confirms its excellent magnetic response (Supplementary Fig. S1f, inset). These results demonstrate the stable and controllable MH performances of the superparamagnetic MRT nanocomposite.

The concentrations of released iron ions in MRT supernatants after magnetic heating treatment (∼50°C) at different pH values were quantified using ICP-OES. Figure 1h indicates the burst release of Fe^2+/3+^ from MRT in an acidic microenvironment under exposure to AMF. In sharp contrast, the final concentration of the released iron ions at pH 7.4 is negligibly as low as 0.11 nM. Additionally, compared with the burst release of iron ions, zinc and manganese ion releases are also negligible even in acidic media (Supplementary Fig. S1g), signifying the relative stability of MRT under MH. Electron spin-resonance (ESR) spectroscopic results in Fig. 1i confirm the generation of •OH radicals via an Fe^2+/3+^-induced Fenton reaction in acidic simulated body fluid (SBF) containing MRT supernatant and H_2_O_2_ after magnetic heating treatment (∼50°C), which is also confirmed by the decreased UV–vis absorbance intensities (664 nm) and the decolorization of methylene blue trihydrate (MB) at decreased pH values due to the produced hydroxyl radicals (Supplementary Fig. S1h). Therefore, these results reveal that MRT nanoparticles can produce abundant hydroxyl radicals in simulated TME (pH = 6.5) due to the sustainable release of Fe ions under the effect of MH, providing an important premise for destroying bacteria in TME.

### *In vitro* antimicrobial and LPS release induced by MRT+MH

For further biological applications, we first evaluated the stability of MRT nanoparticles in SBF solutions. As shown in Supplementary Fig. S1i, no apparent release of iron ions and corresponding decoloration of MB can be found in 52 h of incubation even at pH 6.5 without MH. More importantly, the MH properties of MRT are not significantly affected even if a certain amount of iron ions are released, suggesting a stable and sustainable magnetocaloric performance of MRT (Supplementary Fig. S2). Furthermore, MRT nanoparticles can be well dispersed in PBS and Dulbecco's Modified Eagle's Medium (DMEM) without precipitation for as long as 36 h (Supplementary Fig. S3), which is beneficial for the following biological applications *in vitro* and *in vivo*.

**Figure 2.**
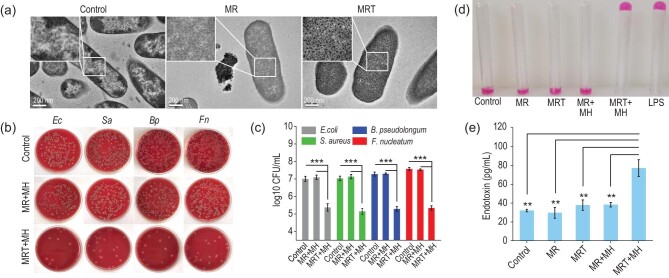
*In vitro* antibiosis and LPS release induced by MRT+MH. (a) Bio-TEM images of *F. nucleatum* (*Fn*) without or with the MR/MRT nanoparticles treatment (400 μg/mL). Scale bar, 200 nm. (b) Representative digital photos of *E. coli* (*Ec*), *S. aureus* (*Sa*), *F. nucleatum* (*Fn*) and *B. pseudolongum* (*Bp*) treated by MR/MRT nanoparticles with MH (MR+MH/MRT+MH) and then incubated by the spread plate method (SPM). (c) The corresponding concentrations of four types of bacteria after the MR+MH or MRT+MH treatment determined by colony counting (*n* = 3). (d) Qualitative detection of endotoxin (LPS) in the supernatant of *Fn* after various treatments by *Limulus* amebocyte lysate assay (0.25 EU/mL). (e) Quantitative concentrations of LPS in the corresponding bacterial supernatants after various treatments by enzyme-linked immunosorbent assay (ELISA) (*n* = 3). Data are expressed as means ± SD (*n* = 3). Statistical significances were calculated via one-way ANOVA, ^*^P < 0.05, ^**^P < 0.01 and ^***^P < 0.001.

It has been reported that positively charged nanoparticles can be spontaneously adsorbed on the negatively charged surface of bacteria through electrostatic attraction [26]. In this work, TPP, a typical kind of positive lipophilic cation ligand, was conjugated on MRT nanoparticles and its specific binding affinity with negative bacterial biofilms enabled MRT to preferentially target onto the bacterial surface. Hence, under exposure to AMF *in vitro*, we investigated the antibacterial effects of MRT against *Fusobacterium nucleatum* (*Fn*), a typical G– in CRC, and three other three types of bacteria including *Escherichia coli* (*Ec*), *Staphylococcus aureus* (*Sa*) and *Bifidobacterium pseudolongum* (*Bp*), which are common bacteria in tumor tissue according to previous reports [27]. From the biological transmission electron microscopic (bio-TEM) images in Fig. 2a, we can see abundant MRT nanoparticles adhering to the surface of bacteria, while MR nanoparticles without TPP grafting are much less visible on the surface of the bacteria, meaning that the designed MRT nanoplatform can be actively adsorbed onto the bacterial surface with high affinity by electrostatic interactions.

Then, the antimicrobial efficacy was evaluated *in vitro*. Under exposure to 1.35 kAm^–1^ AMF, it can be seen that MRT+MH/MR+MH treatments result in a significant temperature rise of *Fn* to ∼50°C, while the pure SBF solution shows a much less significant temperature rise of *Fn*, indicating the good biocompatibility of AMF (Supplementary Fig. S4a). Varied concentrations of MRT without MH exhibit insignificant effects on the growth of various bacteria in the SBF (pH = 6.5) containing 100 μM of H_2_O_2_ (Supplementary Fig. S4b), showing negligible toxicity of MRT nanoparticles against bacteria. Upon exposure to MH, Fenton reagent (Fe^2+^/Fe^3+^) released from MRT nanoparticles will react with the over-expressed H_2_O_2_ in the microenvironment of bacteria-enriched colorectal tumors to efficiently generate •OH radicals in the simulated solution. Therefore, upon MH treatment (∼50^o^C), the viability of bacteria exposed to the MRT nanoparticles decreased significantly under AMF due to the existence of •OH (Supplementary Fig. S4c), indicating that MRT+MH treatment plays an efficient sterilization role under the above conditions. Additionally, bacterial colony counting by the spread plate method (SPM) shows the effective proliferation inhibition of various bacteria in the MRT+MH group compared with other control groups (Fig. 2b). The corresponding quantitative results displayed in Fig. 2c show the much higher antimicrobial efficacy of the MRT+MH group (∼5 log10 CFU/mL) than that of the MR+MH group (∼7 log10 CFU/mL), implying the significant function of the MRT adsorption on the bacterial surface in killing these bacteria. To further prove the indispensable role of hydroxyl radicals in antibiosis, we evaluated the antibacterial efficacy of MRT+MH in the absence of H_2_O_2_. We carried out magnetic heating on four types of bacteria co-incubated with MRT at different concentrations in the simulated TME without H_2_O_2_. As shown in Supplementary Fig. S5a, the survival percentages of bacteria exposed to the MRT nanoparticles at 400 μg/mL are still >70%, and even as high as 93% for *Fn*, confirming that the H_2_O_2_-derived •OH by the Fenton reaction is the main active species in antibiosis.

The biofilms of bacteria are especially susceptible to reactive oxygen species (ROS) attack in the case of magnetic heating due to the promoted permeability of the biofilms, as revealed by bio-TEM imaging. As shown in Supplementary Fig. S5b, we can visually find that the MRT+MH group has disintegrated bacterial biofilms including the cell wall at much higher efficiency than the MR+MH group, indicating strong oxidative damage to the bacteria.

It has been known that bacteria subject to oxidative damage tend to release an endotoxin known as lipopolysaccharide (LPS) externally [28,29]. The impact of MRT+MH on the release of bacterial LPS was evaluated by *Limulus* amebocyte lysate assay via a typical lectin *Limulus* amebocyte lysate, which can sensitively agglutinate with endotoxin to form a stable gel intuitively. As shown in Fig. 2d, only MRT+MH treatment has led to the release of bacterial LPS that reacted with *Limulus* amebocyte lysate to form a stable gel. Subsequently, the LPS concentrations in corresponding bacterial supernatants of various groups were detected using enzyme-linked immunosorbent assay (ELISA). The concentration of released LPS in the MRT+MH group is significantly higher than those of other groups, while simple MH-induced heating to ∼50°C by MR without bacterial targeting can hardly release LPS from *Fn*, further confirming the significant LPS release from damaged *Fn* bacteria by MRT+MH treatment (Fig. 2e). To further investigate the capability of the oxidative-damaged bacteria in regenerating new colonies, we cultured the bacterial precipitates following MRT+MH treatment, which, as we find, could hardly rebind with each other to reform bacteria colonies in 24 h of culture (Supplementary Fig. S5c and d) by SPM. Therefore, MRT+MH treatment can effectively kill the bacteria by ROS generated *in situ* and then lead to the burst release of LPS from the oxidative-damaged bacteria.

### Regulation blockage of *Fn* on CT26 cells by MRT+MH treatment *in vitro*

**Figure 3.**
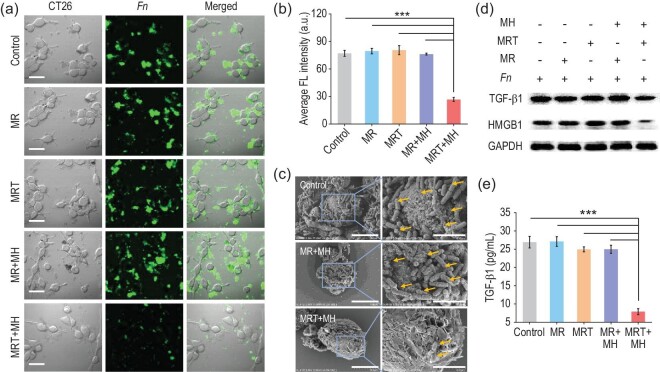
Regulation blockage of *Fn* on CT26 cells by MRT+MH treatment *in vitro*. (a) Representative confocal laser scanning microscopic (CLSM) images of FITC-labeled *Fn* (green) co-cultured with CT26 cells (bright field) after the MR/MRT/MR+MH/MRT+MH treatments. Scale bar, 40 μm. (b) Average fluorescence intensity (FLI) of FITC-labeled *Fn* (green) analysed by ImageJ. (c) Scanning electron microscopy (SEM) images of CT26 cells incubated with *Fn* for 3 h after the MR+MH/MRT+MH treatments. Scale bar, 10 μm (left) and 4 μm (right). (d) Western blotting analyses of TGF-β1 and HMGB1 expressions in CT26 cells incubated with *Fn* after various treatments. (e) TGF-β1 levels determined by ELISA in the supernatants of CT26 cells co-cultured with *Fn* after various treatments. Data are expressed as means ± SD (*n* = 3). Statistical significances were calculated via one-way ANOVA, ^*^P < 0.05, ^**^P < 0.01 and ^***^P < 0.001.

Previous studies have identified that varieties of deleterious bacteria, such as *Fn* adsorbed on the surface of CRC tumor cells, are capable of promoting tumor growth by triggering immunosuppression [30,31]. Hence, we co-cultured *Fn* with a typical mice colon cancer cell (CT26) to mimic a CRC tumor surrounded by bacteria and then explored the effect of *Fn* exposed to various treatments on the growth of CT26 cells *in vitro* using confocal laser scanning microscopic (CLSM) imaging. From Fig. 3a, we can clearly see a large number of fluorescein isothiocyanate (FITC)-labeled *Fn* (green) gathering around the CT26 cells (bright field) in the control group. After being treated with MR, MRT or MR+MH, most *Fn* (green) remained alive, clustering densely around CT26 cells (bright field), while only a minority of *Fn* (green) survived, scattering away from the CT26 cells in the MRT+MH group (Fig. 3a), which is consistent with the CLSM imaging of unlabeled *Fn* after sufficient incubation in Supplementary Fig. S6. Quantitatively, the average green fluorescence intensity (FLI) of FITC-labeled *Fn* is also significantly lower in the MRT+MH group than that of other groups (Fig. 3b). Moreover, the scanning electron microscopy (SEM) images in Fig. 3c display that numerous rod-shaped *Fn* have adhered closely onto the surface of CT26 cells in the control and MR+MH group. In a sharp contrast, upon MRT+MH treatment, significantly fewer bacteria can be seen on the CT26 cell surface, further confirming that the MRT+MH treatment has greatly reduced the number of *Fn* binding to the surface of the tumor cells. By quantitatively detecting the Fe concentration in the supernatants of the *Fn*-CT26 coculture system after various treatments (Supplementary Fig. S7a), 70% of the Fe element has remained in the supernatant of MRT+MH group, demonstrating that most MRT nanoparticles will be resuspended in the supernatant after the destruction of bacteria without significant impact on CT26 cells. Subsequently, as shown in Supplementary Fig. S7b, we find that hot-water incubation for 5 min has no significant effect on the cell viability of CT26 after 24 h, indicating that 50°C alone for 5 min will not effectively inhibit CRC cell (CT26) growth. Neither the MRT+MH-induced transient hyperthermia nor the released Fe^2+^/Fe^3+^ Fenton agent will cause significant antitumoral cytotoxicity. Taken together, these results demonstrate that MRT+MH treatment is capable of exclusively destroying bacteria and preventing their adhesion to cell surfaces without significantly damaging the tumor cells.

TGF-β1 and HMGB1 proteins are closely related to the proliferation and metastasis of cancer cells, and secretory TGF-β1 from tumor cells can inhibit the response of immune cells including DCs and macrophages in TME [32,33]. Therefore, we also evaluated TGF-β1 and HMGB1 expressions of CT26 cells using western blotting (WB) and carried out quantitative calculation of the gray value. These results in Fig. 3d and Supplementary Fig. S8a reveal the greatly lower expression levels of TGF-β1 and HMGB1 in CT26 cells after being incubated with MRT+MH-treated *Fn* than those of other groups. Moreover, under the same experimental conditions, we further quantified the concentration of TGF-β1 in corresponding cell supernatants using ELISA. As shown in Fig. 3e, compared with other treatments, the supernatant of CT26 cells co-cultured with MRT+MH-treated *Fn* contains a markedly lower amount of TGF-β1 than others, implying the largely mitigated immunosuppression caused by TGF-β1 and HMGB1. In addition, the expression of TGF-β1 in CT26 cells incubated with MRT+MH-treated *Fn* is downregulated gradually during prolonged magnetic heating (Supplementary Fig. S8b). These results further manifest that MRT+MH can effectively attenuate the immunosuppressive effect of *Fn* bacteria on CT26 cells due to the *Fn* damage and resultantly the much-inhibited *Fn* binding to CT26 cells.

### LPS released by MRT+MH-treated *Fn* activates antitumor immune responses *in vitro*

As typical PAMPs, exogenous LPS can be recognized by host PRRs to promote local M1 polarization of macrophages and maturation of DCs, thus activating antitumor immune responses [34–36]. To explore whether LPS fragments released from damaged *Fn* after the MRT+MH treatment could indeed activate the immunological system or not, we first collected the supernatants of *Fn* after various treatments to prepare conditioned medium and then investigated the influence of supernatants on the immune behaviors of bone-marrow-derived macrophages (BMDMs) and bone-marrow-derived dendritic cells (BMDCs) by adding these conditioned media into the BMDMs or BMDCs system for 12 h, and commercial LPS was used as a positive control.

**Figure 4.**
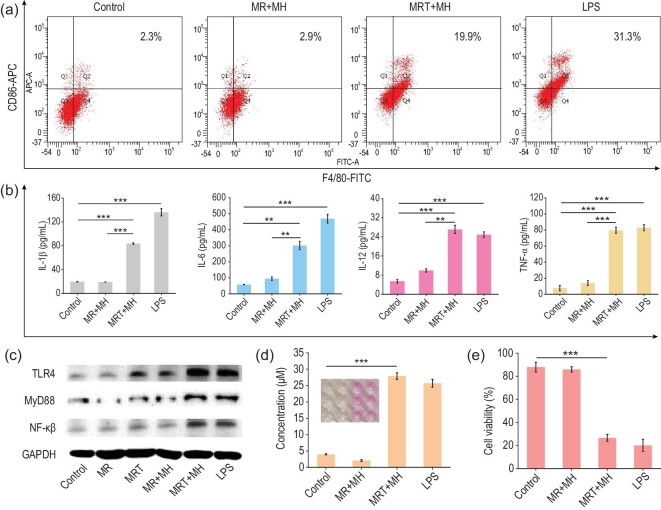
LPS released by MRT+MH-treated *Fn* activates antitumor immune responses *in vitro*. (a) Flow cytometry plots of CD86- and F4/80-positive macrophage (M1 phenotype) fractions of BMDMs after treatment with the conditioned media containing supernatants of *Fn* in different groups, and commercial LPS (10 μg/mL) was used as a positive control. (b) Quantified secretion levels of IL-1β, IL-6, IL-12 and TNF-α in the supernatants of BMDMs after corresponding treatments (*n* = 3). (c) Western blotting analysis of TLR4–MyD88–NF-κB pathway in BMDMs after various treatments *in vitro*. (d) NO secretion of BMDMs detected by Griess assay kit. Inset: digital photos of different groups after reaction (Control, MR+MH, MRT+MH and LPS groups from left to right) (*n* = 3). (e) Viabilities of CT26 cells co-cultured with BMDMs stimulated by the various conditioned media by CCK-8 (*n* = 6). Data are expressed as means ± SD (*n* = 3 or 6). Statistical significances were calculated via one-way ANOVA, ^*^P < 0.05, ^**^P < 0.01 and ^***^P < 0.001.

As shown in Supplementary Fig. S9, the microscopic images clearly show that BMDMs can be stimulated to differentiate into numerous pseudopodia by the conditioned medium containing LPS released from *Fn* after MRT+MH treatment, featuring significantly upregulated CD86 protein, a fundamental M1 marker, as quantitatively manifested by ∼7- and ∼9-fold higher proportions of F4/80^+^CD86^+^ M1 macrophages (19.9%) than that in the MR+MH (2.9%) and control group (2.3%), respectively (Fig. 4a). These results confirm that the release of immunogenic LPS fragments from damaged *Fn* can induce the M1 polarization of macrophages.

The secretion of cytokines and related immune response constitute the fundamentals of the immune system, in which interleukin-1β (IL-1β), interleukin-6 (IL-6) and interleukin-12 (IL-12) are the predominant cytokines in the regulation of immune responses against various diseases [37]. Additionally, M1 polarization of TAMs has been confirmed to play a major role in antitumor innate immune response via mechanisms such as the regulatory effects of various cytokines and macrophage-mediated phagocytosis of tumor cells [38]. Tumor necrosis factor-α (TNF-α) secreted by immune cells is well known to be capable of directly killing tumor cells, while IL-1β triggers can regulate adaptive immune responses via interferon regulatory factor 3 (IRF3) activation and IFN production, presenting a potent immune response against tumor cells [39]. Therefore, it is necessary to validate whether LPS fragments released from damaged *Fn* by MRT+MH could induce the secretions of these cytokines or not. As shown in Fig. 4b, the conditioned medium in the MRT+MH group could distinctly promote the secretions of immunological IL-6, TNF-α, IL-12 and IL-1β cytokines from BMDMs. Subsequently, the TLR4/MyD88/NF-κB signal pathway has been confirmed as the underlying mechanism for activating BMDMs in Fig. 4c, as evidenced by the substantially upregulated expressions of these cytokines in BMDMs once co-incubated with the supernatant containing these LPS fragments. The expression levels of TLR4/MyD88/NF-κB were also analysed using ImageJ (Supplementary Fig. S10), further indicating their upregulation respectively.

It has been reported that nitric oxide (NO) secreted by activated TAMs could act as a cytotoxic and apoptosis-inducing signal molecule against tumor cells [40]. Herein, we quantitatively measured the NO release from BMDMs upon various stimulations using a typical Griess assay. It can be found that BMDMs are able to release NO distinctly once activated by the conditioned medium in the MRT+MH group and correspondingly the Griess reagent also changes from colorless into light red after reacting with NO in the MRT+MH group (Fig. 4d). Then we evaluated the performance of M1-phenotype BMDMs in killing tumor cells using the typical cell-counting kit 8 (CCK-8) assay. As shown in Fig. 4e, the viability of CT26 cells co-cultured with BMDMs under stimulation by the conditioned medium containing LPS fragments in the MRT+MH group is as low as ∼27%, in comparison to those of other control groups (>80%), confirming the excellent tumoral cytotoxicity of M1 macrophages. Finally, the M2-to-M1 phenotype transformation of BMDMs stimulated using the conditioned medium containing LPS fragments in the MRT+MH group was also quantitatively detected by flow cytometry using the M2 biomarker CD206. As shown in Supplementary Fig. S11, the percentage of F4/80^+^CD206^+^ M2 macrophages in the MRT+MH group is significantly lower than those of the MR+MH and control group, further manifesting the M2-to-M1 subtype transformation of BMDMs by the released LPS fragments. All these results demonstrate that the LPS fragments released from MRT+MH-treated *Fn* are capable of activating the macrophages into M1 polarization via the TLR4/MyD88/NF-κB pathway, which results in significant secretions of various cytokines against tumor cells *in vitro*.

Dendritic cells (DCs), as a kind of fundamental antigen-presenting cell, play critical roles in effectively initiating the adaptive immunity [41]. The CD80 and CD86 (co-stimulation molecules), as well as the major histocompatibility complex class II (MHC II) molecules symbolize the maturation and antigen presentation level of DCs for adaptive immune activation [42]. We then investigated the maturation and antigen presentation of BMDCs following various stimulation *in vitro*. First, we observed the morphological changes of BMDCs stimulated by the conditioned medium containing supernatants of *Fn* by microscopic imaging (Supplementary Fig. S12). Obviously, BMDCs can be remarkably stimulated to differentiate into dendritic pseudopodia in the MRT+MH group, qualitatively signifying the maturation of DCs stimulated by the LPS fragments released from MRT+MH-treated *Fn*.

In order to verify the maturation and antigen presentation of DCs, CD80, CD86 and MHC II antibodies were detected using flow cytometry. As displayed in Supplementary Fig. S13a, the percentage of CD80^+^CD86^+^ BMDCs treated by LPS fragments from the MRT+MH group (31.55%) is significantly higher than those in the control (9.74%) and MR+MH (7.66%) group. Meanwhile, LPS fragments released from MRT+MH-treated *Fn* also elevate the expression of MHC II in BMDCs (Supplementary Fig. S13b), indicating the enhanced antigen presentation of BMDCs once in contact with free LPS fragments. Taken together, these results corroborate that *in vitro* LPS fragments released from MRT+MH-treated *Fn* can not only significantly stimulate the maturation of DCs, but also effectively enhance the antigen presentation of DCs, providing an important prerequisite for inducing a T-cell immune response.

### *In vivo* anticancer efficacy evaluation of MRT+MH

**Figure 5.**
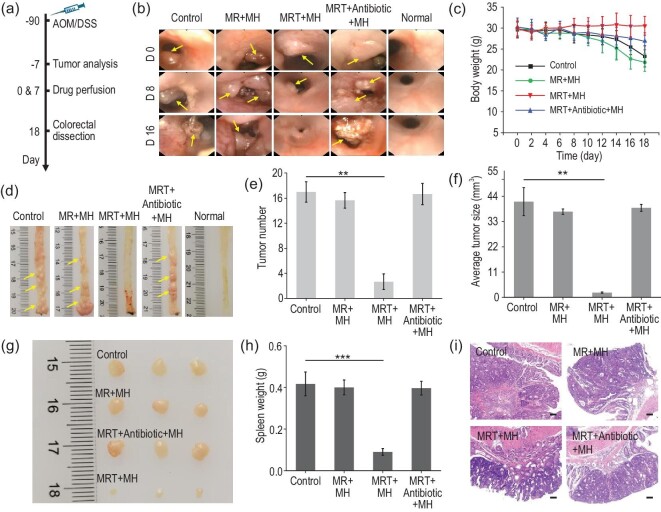
*In vivo* anticancer efficacy evaluation of MRT+MH. (a) Schematic diagram of experimental design for the establishment and treatment of an orthotopic colorectal cancer (CRC) model (*n* = 10). (b) Representative colonoscopy photos of CRC mice in Control/MR+MH/MRT+MH/MRT+Antibiotic+MH/Normal groups on designated days (Days 0, 8 and 16) (*n* = 6 or 7). Yellow arrows indicate the tumor tissues. (c) Time-dependent body weight surveillance of mice for 18 days after various administrations (*n* = 4 or 6). (d) Macroscopic morphologies of dissected colorectal tissues in CRC mice at the end of treatments (*n* = 4). Yellow arrows indicate the tumor tissues. (e) Tumor number and (f) average tumor size per colon in mice at the end of treatments (*n* = 4). (g) Digital photos of the largest harvested tumors per colon (*n* = 3). (h) Weight of dissected spleen tissues after different treatments (*n* = 4). (i) Representative images of H&E staining of the distal colon in various groups. Scale bar, 100 μm. Data are expressed as means ± SD (*n* = 4 or 6). Statistical significances were calculated via one-way ANOVA, ^*^P < 0.05, ^**^P < 0.01 and ^***^P < 0.001.

Encouraged by the noticeable immune response activation by MRT+MH *in vitro*, we then further evaluated its therapeutic efficacy and antitumor immune responses on an orthotopic CRC model, which could more truly simulate the development of cancer and its tissue microenvironment. As illustrated in Fig. 5a, the orthotopic colorectal tumors on male C57BL/6 mice were induced by chemical carcinogens named azoxymethane (AOM) and dextran sodium sulfate (DSS) [43]. Seven days before administration, we confirmed cancerization in the colorectal region of mice through hematoxylin & eosin (H&E) staining of the dissected colorectal tissues. As shown in Supplementary Fig. S14a, the AOM/DSS-stimulated mice have exaggerated crypt damage and obvious adenomatous polyps compared with the normal mice, which are typical of intestinal tissue canceration. Correspondingly, we also counted the tumor number (approximately seven) and measured the tumor size (∼11 mm^3^) in dissected colorectal tissues of the CRC model and the colorectal part of the CRC mice model is significantly shortened in the digital photo (Supplementary Fig. S14b). Therefore, the AOM/DSS chemical method can successfully induce an orthotopic CRC mice model.

Since an infrared camera could not detect the temperature change of deep tissues such as the intestine in mice, we initially injected the same concentration of MR/MRT nanoparticles subcutaneously into mice abdomen and then conducted MH for 5 min under exposure to AMF (1.35 kAm^–1^). By monitoring the infrared camera, it could be observed that the temperature of the injection site rose to ∼50°C quickly in the MR/MRT group while the PBS group still maintained normal body temperature (Supplementary Fig. S15a). Moreover, the MH behavior further confirms the excellent magnetothermal stability of MR/MRT *in vivo* under AMF, which could be sustainably maintained at ∼50°C (Supplementary Fig. S15b). Subsequently, to prevent the rapid heating-up of the metal-based thermocouple probe during AMF, we used the thermocouple thermometer to detect the temperature change at the designated site of the distal colon immediately after turning off AMF, which showed a temperature loss of ∼6.5°C during the period. As displayed in Supplementary Fig. S15c, the temperature of the corresponding colorectal region could reach ∼50.6°C (44.1°C + 6.5°C) under AMF for 5 min upon rectal perfusion of MRT nanoparticles. Importantly, as shown in the bioluminescence images of MRT nanoparticles after intestinal perfusion (Supplementary Fig. S16), MRT is effectively enriched in the colorectal region of mice within 1 h but will be completely excreted in feces outside of the body in 24 h, indicating the excellent biosafety of perfused MRT nanoparticles after performing the therapy on the colorectal tissue.

Afterwards, orthotopic colorectal-tumor-bearing mice were randomly divided into four groups: Control, MR+MH, MRT+MH and MRT+Antibiotic+MH groups (*n* = 10). Intestinal perfusion was used for the administration. In order to better evaluate the therapeutic effect, we also used intestinal photographs of healthy mice as a reference. As shown in Fig. 5a, the perfusion administration and MH treatment were conducted twice with an interval of 7 days and, during the treatment, we used colonoscopy imaging to evaluate the development of CRC at Days 0/8/16. In Fig. 5b, the colonoscopy images clearly reveal the presence of tumors (yellow arrows) in the distal colon of CRC mice. Compared with the normal mice, CRC developed significantly more rapidly in the PBS-treated control group, featuring severe inflammation, intestinal bleeding and abnormal number and size of tumors in the distal colon, demonstrating the high malignancy degree of the tumor. Moreover, tumors in the MR+MH group also grew rapidly, demonstrating that even magnetic heating up to 50^o^C could not inhibit tumor growth in the absence of the bacterial-targeting function of the MR nanoparticles. In sharp contrast, MRT+MH treatment almost eliminated the tumor completely in the colorectal tissue of CRC mice despite slight inflammation, indicating that nanocatalytic ablation of targeted bacteria in CRC under magnetic heating treatment can effectively inhibit tumor growth. In the MRT+Antibiotic+MH group, the intestinal tracts were first infused with bacteria-targeting MRT nanocatalyst, then flushed with antibiotics to remove the bacteria and finally an MH treatment was applied. The corresponding tumor growth was found to be almost unaffected, further indicating that only nanocatalytic ablation of bacteria by ROS produced from the MRT+MH-mediated Fenton reaction, rather than the ROS alone from the MRT+MH-induced Fenton reaction, could perform significant inhibition of tumor growth.

Meanwhile, the weights of the mice in each group were recorded every 2 days. As can be seen in Fig. 5c, CRC mice upon MRT+MH treatment show well-maintained body weights, while those in other groups show much decreased body weights due to severe hematochezia and laxity. At the end of treatment, the harvested colorectal tissues of the MRT+MH-treated mice display strikingly reduced tumor numbers (yellow arrows) and thinner bowel wall compared with other groups (Fig. 5d). Tumor numbers per colon in the Control, MR+MH and MRT+Antibiotic+MH groups (17, 15.7 and 16.7, respectively) are significantly higher than that (2.7) of the MRT+MH group (Fig. 5e). Correspondingly, the average tumor size per colon of the MRT+MH group (2.06 mm^3^) is about one-twentieth of those in the other three groups on Day 18 (Fig. 5f). The digital photos of the largest tumors dissected from the distal colon further show a far smaller size of the tumors in the MRT+MH group in sharp contrast to the other treatments (Fig. 5g), showing significant tumor inhibition by the MRT+MH treatment.

Furthermore, compared with the CRC mice treated with MRT+MH, those in the other three groups had much enlarged spleen tissues (Fig. 5h and Supplementary Fig. S17)—a kind of concurrent pathological phenomenon caused by CRC [44]. Meanwhile, pathological analysis of colon tissues in the different groups was also conducted using H&E staining. As shown in Fig. 5i and Supplementary Fig. S18, there are obviously damaged cyrpts (U-shaped structures), goblet cell (goblet-shaped) loss and tumor protrusion on the glands of the colon along with the intense infiltration of mucosa in the Control, MR+MH and MRT+Antibiotic+MH groups, while these pathological phenomena of colon tissues are almost absent in the MRT+MH-treated group. Taken together, all these results verify the outstanding antitumor efficacy of MRT+MH treatment depending on the MRT+MH-induced bacteria disintegration in the CRC, instead of a simple magnetocaloric effect or oxidative damage by Fenton reaction-produced ROS.

### Mechanism of anticancer immune activations *in vivo*

The notorious immunological tolerance and drug resistance in CRC is mainly attributed to enrichment of microbiome in the TME, especially the non-negligible *Fn* [45]. To achieve systematic anticancer immune response activation, the first crucial step is to effectively disintegrate bacteria and release LPS as PAMPs in the TME of CRC, as demonstrated by the *in vitro* results. Thus, we performed 16S rRNA high-throughput sequencing to elucidate the effect of MRT+MH on the bacterial taxonomic proportion in the mice feces. As we know, α- and β-diversities are the fundamental components in microbial taxonomy evaluation of fece samples, which manifest the change in microbial composition in intestinal tissues.

The α-diversity of intestinal bacteria represented by Chao1 and ACE is decreased significantly due to the development of CRC in control groups, while MRT+MH treatment can restore the microbial diversity close to a normal steady state (Supplementary Fig. S19a and b). Weighted unifrac analysis, known as β-diversity, reveals that the development of CRC in the control groups will also induce a marked difference in gut microbiota composition between CRC-bearing mice and healthy ones, whereas MRT+MH treatment closes such a difference to a large extent (Supplementary Fig. S19c). Furthermore, MRT+MH treatment keeps the relative abundance of *Bacteroidota* or *Firmicutes* at a much-lowered level, close to the normal gut in healthy mice, and correspondingly reduces the *Fn* load in the harvested colorectal tissues remarkably compared with other treatments (Supplementary Fig. S19d and e). On the other hand, we also detected the level of LPS in the serum to confirm whether the LPS fragments are released from the bacteria by the treatments into the systemic circulation or not. ELISA analysis shows that the MRT+MH-treated mice had a greatly higher concentration of LPS in the serum than other groups (Supplementary Fig. S19f). Together, these results demonstrate that MRT+MH treatment can effectively rectify the abnormal diversity of intestinal microbiota in CRC mice. Especially, such a treatment is capable of disintegrating pathogenic bacteria in CRC such as *Fn*, which, more attractively, leads to LPS fragment release as PAMPs for the immune activations, both innate and adaptive, as verified in the following.

**Figure 6.**
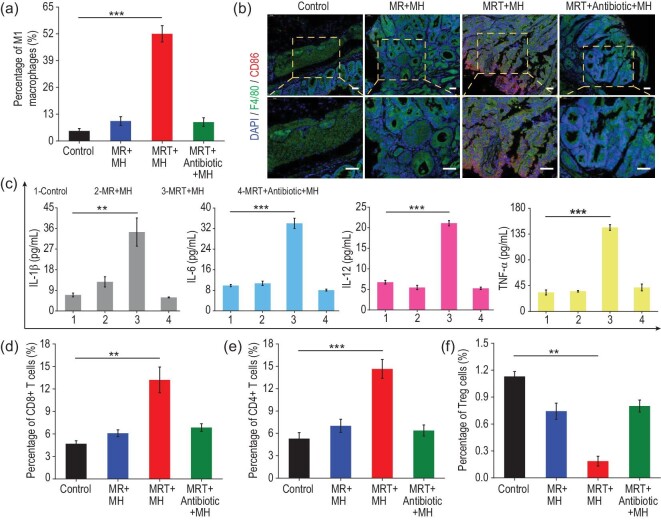
Mechanism of anticancer immune activations *in vivo*. (a) Quantitative analysis of the percentages of M1 macrophages (marked with F4/80^+^CD86^+^) in the harvested colorectal tumor tissues (*n* = 3). (b) Representative images of F4/80 (green) and CD86 (red) co-stained colorectal tissues collected from CRC-bearing mice after various treatments; the nucleus was counter-stained with 4^′^,6-diamidino-2-phenylindole (DAPI) (blue). Scale bar, 50 μm. (c) Quantifications of the secretion levels of cytokines IL-1β, IL-6, IL-12 and TNF-α in the serum isolated from the different groups by ELISA (*n* = 3). (d–f) Quantitative analysis of the percentages of (d) CD8^+^ T cells, (e) CD4^+^ T cells and (f) Tregs cells (*n* = 3). Data are expressed as means ± SD (*n* = 3). Statistical significances were calculated via one-way ANOVA, ^*^P < 0.05, ^**^P < 0.01 and ^***^P < 0.001.

To further explore the underlying mechanism of the antitumor immune response activation by MRT+MH treatment, immune cells in the collected colorectal and spleen tissues were assessed on the 10th day after the first treatment. The percentage of M1 macrophages represented by F4/80^+^CD86^+^ was quantitatively evaluated using flow cytometry in the harvested tumors and the proportion of M1 macrophages in the MRT+MH group was ∼52%, showing a more than 7–13 times increase compared with the Control/MR+MH/MRT+Antibiotic+MH group (3.70%, 7.71% and 7.25%, respectively) (Fig. 6a and Supplementary Fig. S20). In addition, the colorectal tissues of different groups were also collected to validate the intratumoral presence of M1 macrophages using immunofluorescence staining. F4/80 (green) and CD86 (red) were used to label all and M1-phenotype macrophages, respectively. The immunofluorescence results qualitatively reveal that the MRT+MH treatment significantly increases the amount of M1 macrophages featuring a greatly intensified CD86 fluorescence signal (Fig. 6b). These results indicate that *in vivo* MRT+MH treatment is capable of activating TAMs into M1 phenotype depending on bacterial disintegration by MRT+MH.

Subsequently, we attempted to analyse the corresponding cytokines in the serum and T cells in spleen tissues of different treatment groups to further identify the potential activation of adaptive immunity post various treatments. Secreted cytokines, such as IL-1β, TNF-α and IL-6, which are typically related to immediate immune responses against diseases, were evaluated using ELISA in the isolated serum from different treatment groups. As shown in Fig. 6c, the levels of these cytokines are greatly higher in the serum isolated from MRT+MH-treated mice than in other groups, indicating successful activation of antitumor immune response *in vivo*.

More attractively, adaptive antitumor immunity featuring T effector lymphocytes plays an even more important role in the immune response against tumors in cancer immunotherapy, which typically includes cytotoxic T cells (CD8^+^ T cells), T helper cells (CD4^+^ T cells: CD3^+^CD4^+^) and regulatory T cells (Tregs: CD4^+^CD25^+^Foxp3^+^) [46]. First, CD8^+^ T cells in spleen were detected using flow cytometry, which can directly cause the apoptosis of tumor cells and are closely associated with the differentiation of effector memory T cells [47]. As expected, CRC mice after MRT+MH treatment exhibit the highest percentage of CD8^+^ T cells (13.21%), which is ∼2.6-, ∼2.2- and ∼1.9-fold higher than that in the Control/MR+MH/MRT+Antibiotic+MH group, respectively (Fig. 6d and Supplementary Fig. S21), addressing the significant immune mechanism of sustainable and excellent antitumor effects in MRT+MH-treated CRC mice. Additionally, the proportion of CD4^+^ T helper cells that can help to maintain antitumor immunity also had a significant increase in the MRT+MH-treated group (14.64%) compared with the other three groups (∼5%, ∼7% and ∼6% for the Control/MR+MH/MRT+Antibiotic+MH group, respectively) (Fig. 6e and Supplementary Fig. S22). On the other hand, Tregs that counteracts the antitumor responses of T effector cells were also investigated and the percentages of CD25^+^Foxp3^+^ Tregs in the spleen (Fig. 6f and Supplementary Fig. S23) after MRT+MH treatment show a marked decrease in comparison to the control groups, implying the much-inhibited immune suppressive effect of Tregs and the correspondingly enhanced antitumor immunity. All these results clearly validate that antitumor immunity, both innate and adaptive, in CRC mice can be reactivated by MRT+MH treatment through bacteria-reinforced tumor immunosuppression removal and the LPS release by magnetic-promoted nanocatalytic-induced bacteria damage.

Hematological and histological safeties of MRT+MH treatment were also investigated. First, the magnetic heating performances of physiological buffers like PBS and DMEM were evaluated under AMF at 1.35 kAm^–1^, which hardly causes a significant temperature rise (<35°C) (Supplementary Fig. S24), implying the biocompatibility of MH *in vivo*. Notably, all the hematological parameters of MRT+MH-treated mice show no distinct fluctuations after feeding for 5/10/15 days (Supplementary Figs S25 and S26). H&E staining of major organs (hearts, livers, spleens, lungs and kidneys) harvested from MRT+MH-treated mice displayed no significant tissue abnormality after 5/10/15 days (Supplementary Fig. S27), further verifying the excellent compatibility and negligible side effect of MRT+MH treatment.

## DISCUSSION

In summary, we report here a bacteria disintegration-promoted nanocatalytic immune activation strategy for efficient anticancer immunotherapy by the oxidative damage-induced release of lipopolysaccharide, a typical pathogen-associated molecular pattern, from damaged bacteria under MH, the opposite of tumor protection against treatments by TAB. Briefly, the synthesized MRT nanoparticles show a positive-charged surface and excellent MH performance, which enables highly selective and efficient accumulation on the negatively charged surface of TAB coated on solid tumors. The subsequent magnetic heating-promoted Fenton reaction catalysed by the iron ions released from the MRT nanoparticles disintegrates bacterial biofilms via *in situ* oxidative damage, leading to significant LPS release from the damaged bacteria, which acts as immunogenic PAMPs to activate antitumor immunity, including innate macrophage polarization into antitumor M1 phenotype and maturation of DCs via the TLR4–MyD88–NF-κB pathway and the consequent adaptive T effector cells awakening, finally achieving excellent antitumor immunotherapeutic efficacy in the orthotopic CRC mice model. The proposed bacteria-promoted nanocatalytic tumor immunity activation is expected to be a clinically promising therapeutic modality for cancer integrated immunotherapy without using any highly toxic chemodrug, especially for intractable CRC in which the abundant colonization of bacteria such as *Fn* in tumor tissue causes drug resistance and immunosuppression of tumor cells.

## METHODS

The detailed experimental reagents and methods can be found in the Supplementary information file.

### Statistical analysis

Data for *n* ≥ 3 independent experiments were expressed as mean ± standard deviation (SD). Average fluorescence intensity of CLSM images and integrated density of WB results are analysed using ImageJ (version 1.8.0). The statistical significances in this work were analysed via a one-way analysis of variance (ANOVA) using the software GraphPad Prism (version 8.0), n.s., ^*^P < 0.05, significant; ^**^P < 0.01, moderately significant; ^***^P < 0.001, highly significant.

### Ethical statement

All *in vivo* experimental procedures were approved and followed the guidelines for the Animal Care Ethics Commission of Shanghai Tenth People's Hospital, Tongji University School of Medicine (ID: SHDSYY-2018-Z0026).

## Supplementary Material

nwac169_Supplemental_File
