# Supplementary material for: Nanocatalytic bacteria disintegration reverses immunosuppression of colorectal cancer
Source: Natl Sci Rev. 2022 Aug 29;9(11):nwac169. doi: 10.1093/nsr/nwac169 (PMC9647001; doi:10.1093/nsr/nwac169)
Supplement: nwac169_Supplemental_File [file nwac169_supplemental_file.pdf]

Supplementary Information

Nanocatalytic bacteria disintegration reverses immunosuppression of colorectal cancer

Han Jiang[1,2], Yuedong Guo[1,2], Zhiguo Yu[1,2], Ping Hu[1,3*] and Jianlin Shi[1,2,3,*]

[1]State Key Laboratory of High Performance Ceramics and Superfine Microstructures, Shanghai Institute of Ceramics, Chinese Academy of Sciences; Research Unit of Nanocatalytic Medicine in Specific Therapy for Serious Disease, Chinese Academy of Medical Sciences, Shanghai 200050, China;

[2]Center of Materials Science and Optoelectronics Engineering, University of Chinese Academy of Sciences, Beijing 100049, China;

[3]Shanghai Tenth People's Hospital, Shanghai Frontiers Science Center of Nanocatalytic Medicine, School of Medicine, Tongji University, Shanghai 200092, China

***Corresponding authors**. E-mails: huping@mail.sic.ac.cn; jlshi@mail.sic.ac.cn

1. Supplementary Figures

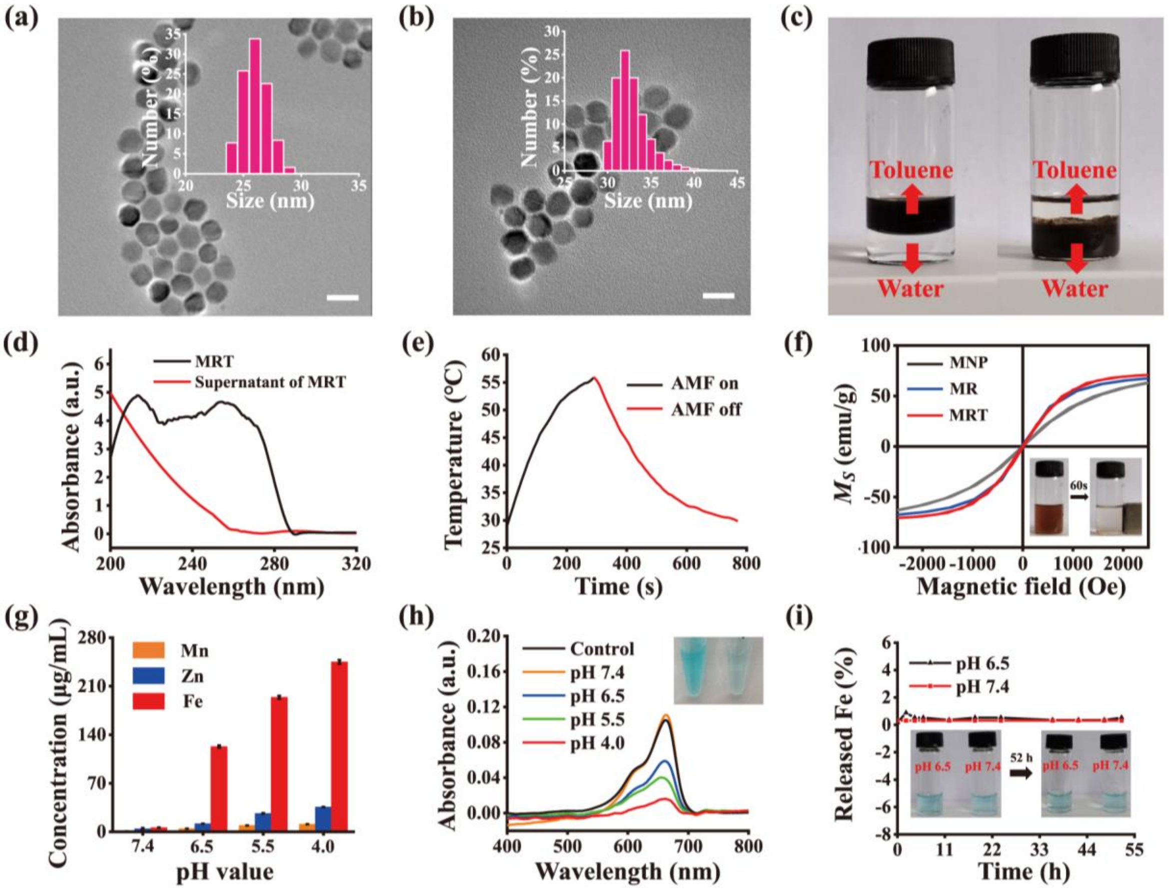

Figure S1. TEM images of (a) MNP in cyclohexane and (b) MR in ethanol; Inset: DLS size distributions. Scale bar, 20 nm. (c) Representative digital photos of MNP dispersed in toluene (left) and MR dispersed in deionized water (right). (d) The UV-vis spectra of RGD on the surface of MRT dispersed in deionized water before and after centrifugation. (e) Magnetic hyperthermia (MH) performance of MRT nanoparticles dispersed in aqueous solution (4 mg/mL) under AMF (H = 1.35 kAm^{-1}, B = 1.7 mT) for a period. (f) Field-dependent magnetization hysteresis loop of MNP/MR/MRT nanoparticles at 300 K. Inset: Photograph showing the ferrofluidic behavior of MRT nanoparticles after dispersion in PBS for 60 s. (g) The pH-dependent Fe/Mn/Zn releases from MRT (4 mg/mL) after MH in SBF at varied pH values (n = 3). (h) UV-vis absorbance spectra of MB monitored at 664 nm when the supernatants of MRT after MH were added into MB solution containing H_2O_2, pH = 4.0/5.5/6.5/7.4). Inset: digital photos of MB before (left) and after (right) the additions of MRT supernatants after MH exposure in the presence of H_2O_2, pH =6.5. (i) *In vitro* the release of Fe from MRT nanoparticles in SBF solutions (pH = 6.5/7.4) at room temperature (no MH treatment). Inset: digital photos of MB solutions upon the additions of MRT supernatants for 52 h in the presence of H_2O_2 (no MH treatment). Data are expressed as means ± SD (n = 3).

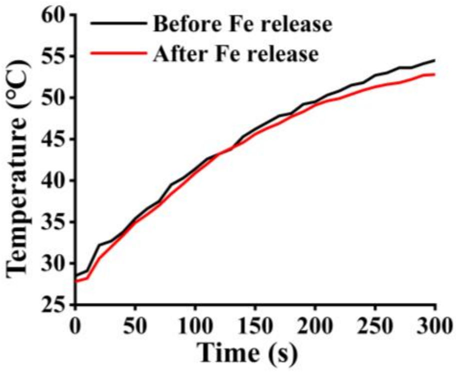

Figure S2. The temperature-rising curves of MRT nanoparticles (4 mg/mL) under MH exposure before and after Fe release.

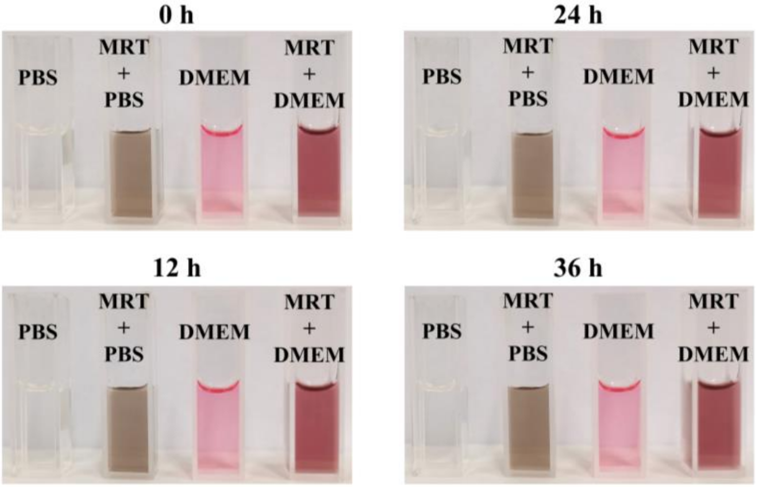

Figure S3. Digital photos of MRT nanoparticles in PBS and DMEM solutions during a period of 36 h (0/12/24/36 h);

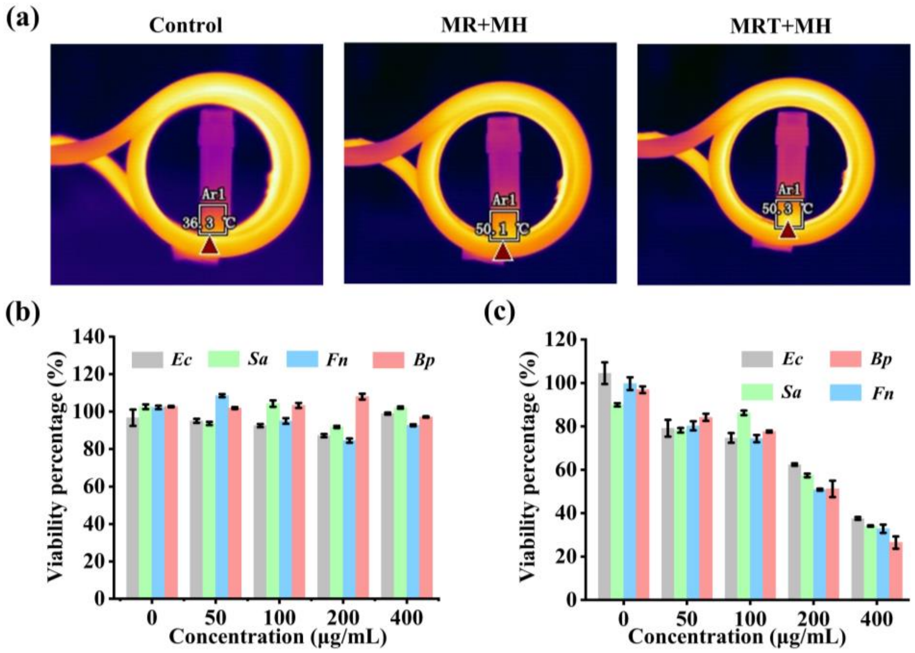

Figure S4. (a) Real-time *in vitro* infrared (IR) thermal imaging of *Fn* treated by MR/MRT under MH exposure (MR+MH or MRT+MH) in the SBF (pH = 6.5). (b) Cytotoxicity of MRT nanoparticles (0, 50, 100, 200 and 400 μg/mL) without MH against various bacteria (*Ec*, *Sa*, *Fn* and *Bp*) according to the OD_{600} in the SBF (pH = 6.5) containing H_2O_2 (100 μM) (n = 6). (c) Cytotoxicity of MRT nanoparticles (0, 50, 100, 200 and 400 μg/mL) under MH exposure against various bacteria (*Ec*, *Sa*, *Fn* and *Bp*) according to the OD_{600} in the SBF (pH = 6.5) containing H_2O_2 (100 μM) (n = 6). Data are expressed as means ± SD (n = 6).

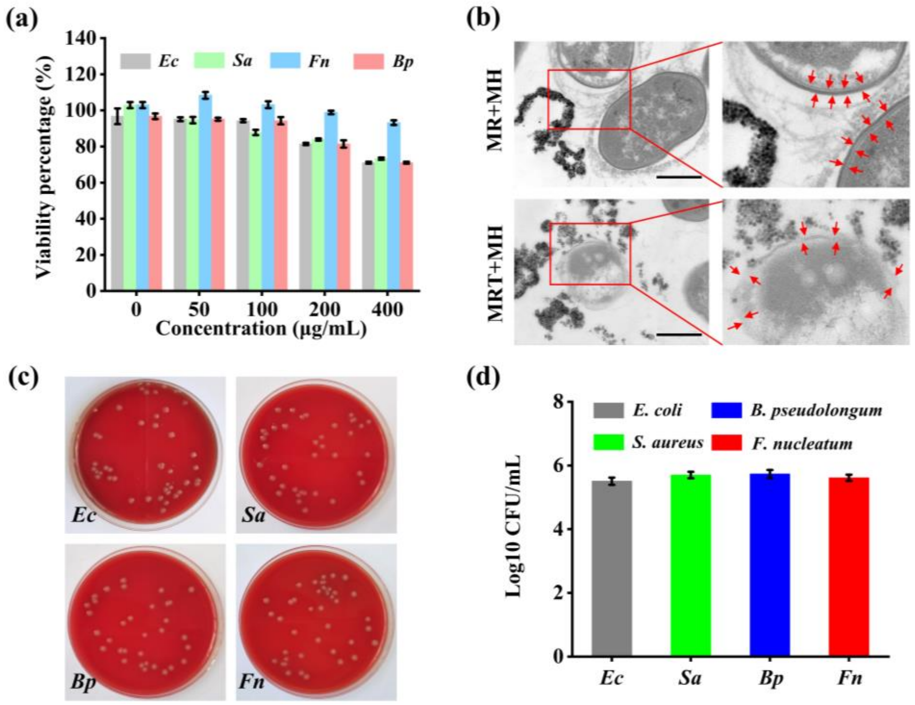

Figure S5. (a) Cytotoxicity of MRT nanoparticles (0, 50, 100, 200 and 400 μg/mL) under MH exposure against various bacteria (*Ec*, *Sa*, *Fn* and *Bp*) according to the OD_{600} in the SBF (pH = 6.5) without H_2O_2 (n = 6). (b) Bio-TEM images of *F. nucleatum* (*Fn*) treated by MR/MRT nanoparticles (400 μg/mL) with MH. Scale bar, 500 nm. Red arrows indicate the bacterial biofilms. (c) Representative digital photos of *Fn* after the MRT+MH treatment when anaerobically incubated by the spread plate method (SPM) for 24 h. (d) The corresponding concentrations of four bacteria after the MRT+MH treatment determined by colony counting results (n = 3). Data are expressed as means ± SD (n = 3 or 6). Statistical significances were calculated via one-way ANOVA, *$p < 0.05$, **$p < 0.01$ and ***$p < 0.001$.

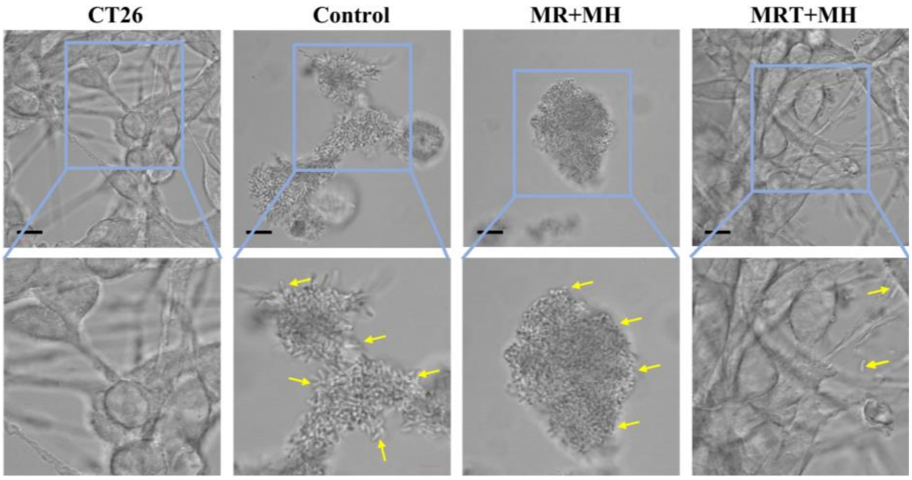

Figure S6. The CLSM images of CT26 cells cocultured with unlabeled *Fn* (yellow arrows, rod-like) after different treatments for 6 h. Scale bar, 10 μm.

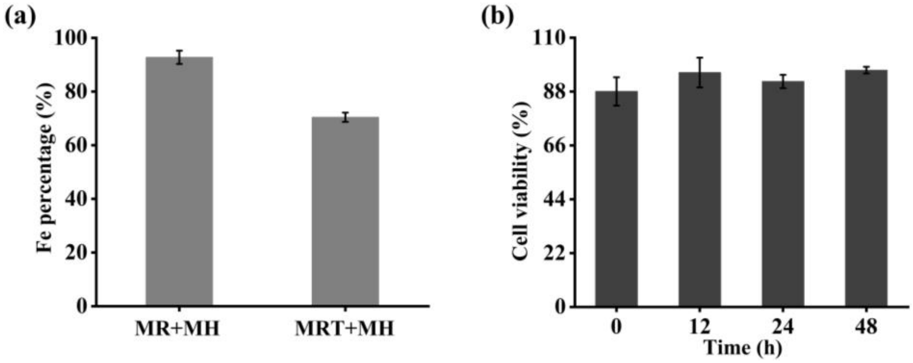

Figure S7. (a) The percentage of Fe element detected by ICP-OES in the supernatants of *Fn*-CT26 coculture system after MR+MH/MRT+MH treatments. (b) The cell viability of CT26 during 0-48 h after water bath at 50 ℃ for 5 min.

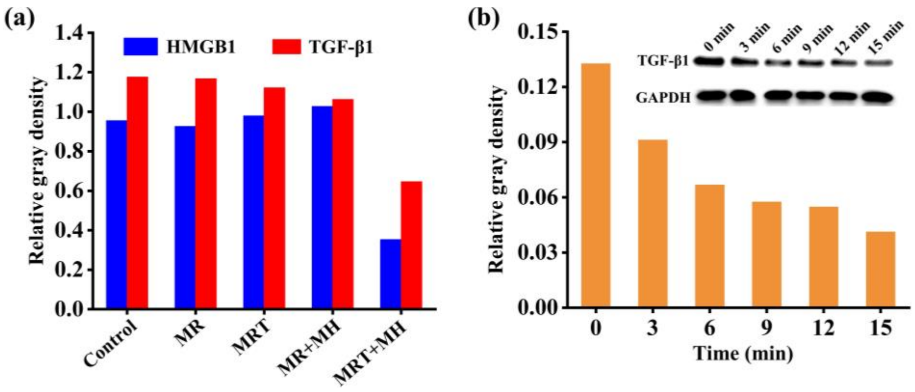

Figure S8. (a) Gray value analysis of TGF-β1 and HMGB1 in CT26 cells when co-incubated with *Fn* after various treatments according to the corresponding WB result, MH for 5 min. (b) WB result and corresponding gray value analysis of TGF-β1 in CT26 cells when co-incubated with *Fn* after MRT+MH, MH for 0-15min.

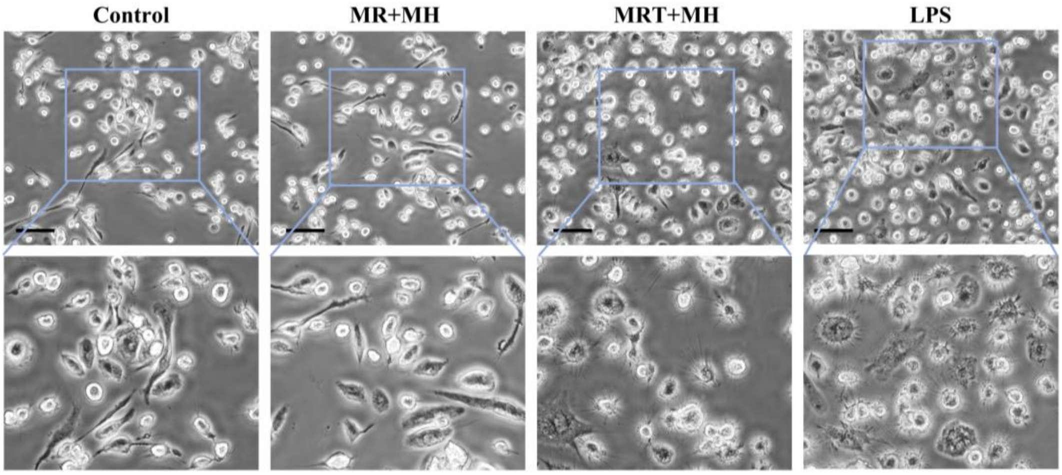

Figure S9. Representative microscopic images of BMDMs stimulated by the conditioned media containing the supernatants of *Fn* after various treatments, and commercial LPS (10 μg/mL) was used as a positive control. Scale bar, 300 μm.

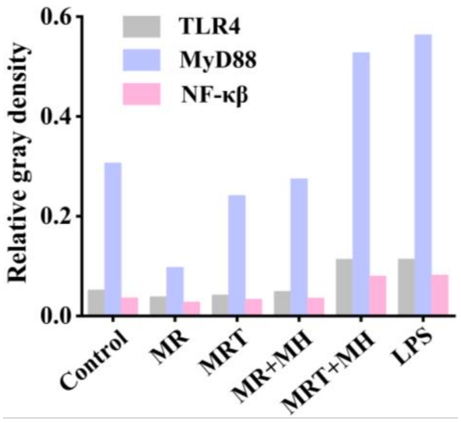

Figure S10. Quantitative analysis of TLR4/MyD88/NF-κB proteins in BMDMs after various stimulations according to the corresponding WB result by ImageJ.

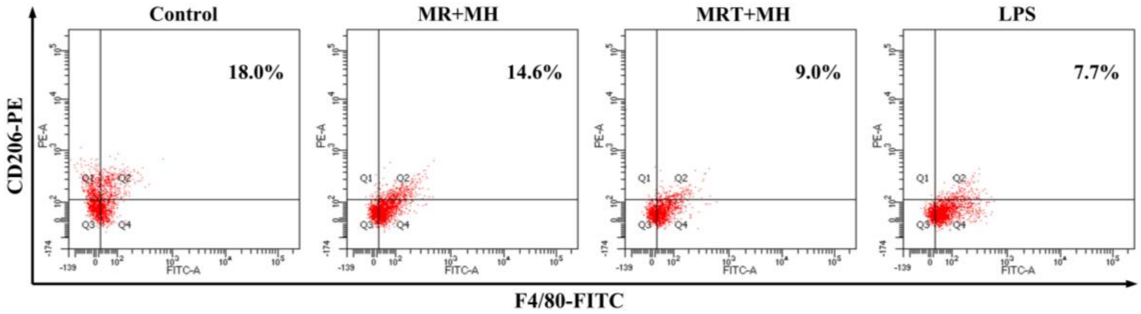

Figure S11. Flow cytometry evaluating the percentages of M2 BMDMs ($CD206^{+}F4/80^{+}$) stimulated by the conditioned media in various groups, and commercial LPS (10 µg/mL) as positive control.

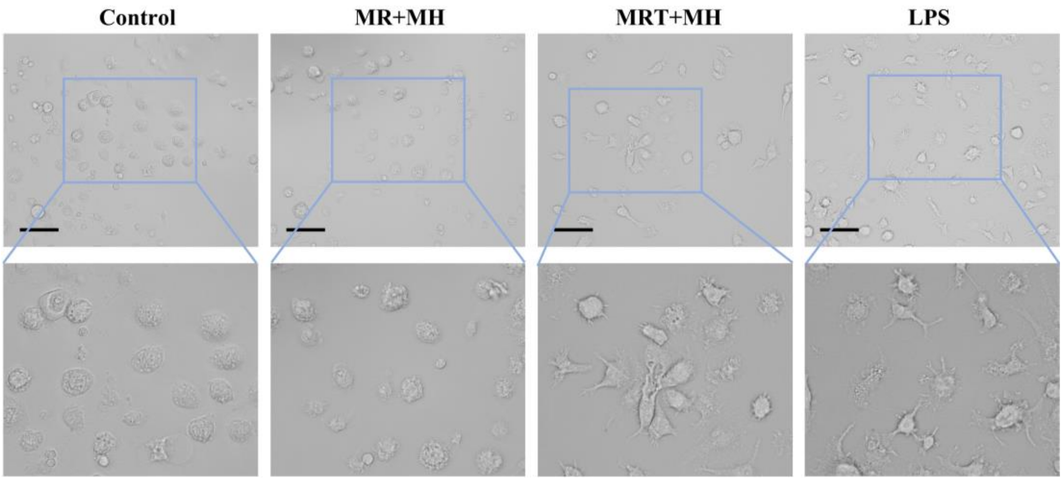

Figure S12. Representative microscopic images of BMDCs stimulated by the conditioned media containing the supernatants of *Fn* after various treatments. Scale bar, 125 µm.

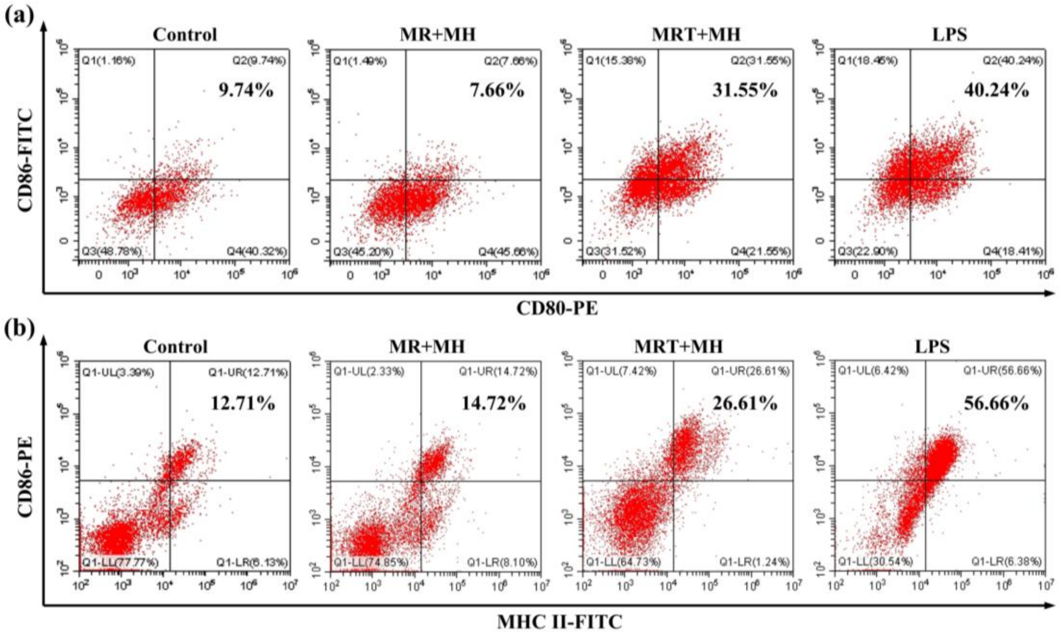

Figure S13. Representative flow cytometry plots indicate the proportions of (a) $CD80^{+}CD86^{+}$ and (b) $CD86^{+}MHC\ II^{+}$ BMDCs in four groups. The BMDCs in four groups were stimulated by the conditioned media containing the supernatants of *Fn* after various treatments for 12 h.

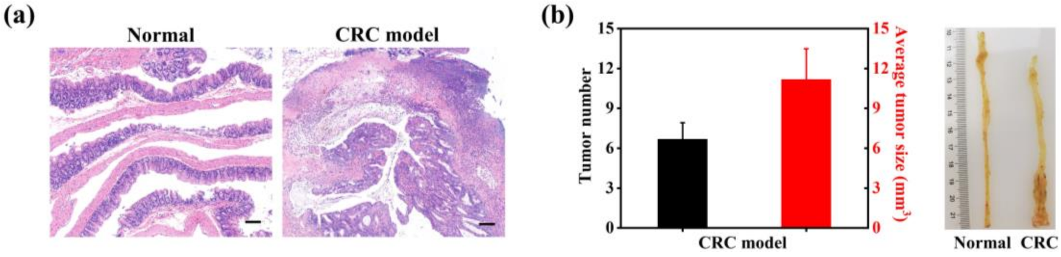

Figure S14. (a) Representative images of Hematoxylin & Eosin (H&E) stained colorectal tissues excised from normal and CRC mice on the 83th day after AOM/DSS administration. Scale bar, 100 μm. (b) Average tumor number and size per colon in CRC model on the 83th day after AOM/DSS administration (n = 3). Inset: representative photos of harvested colorectal tissues from normal and CRC mice. Data are expressed as means ± SD (n = 3).

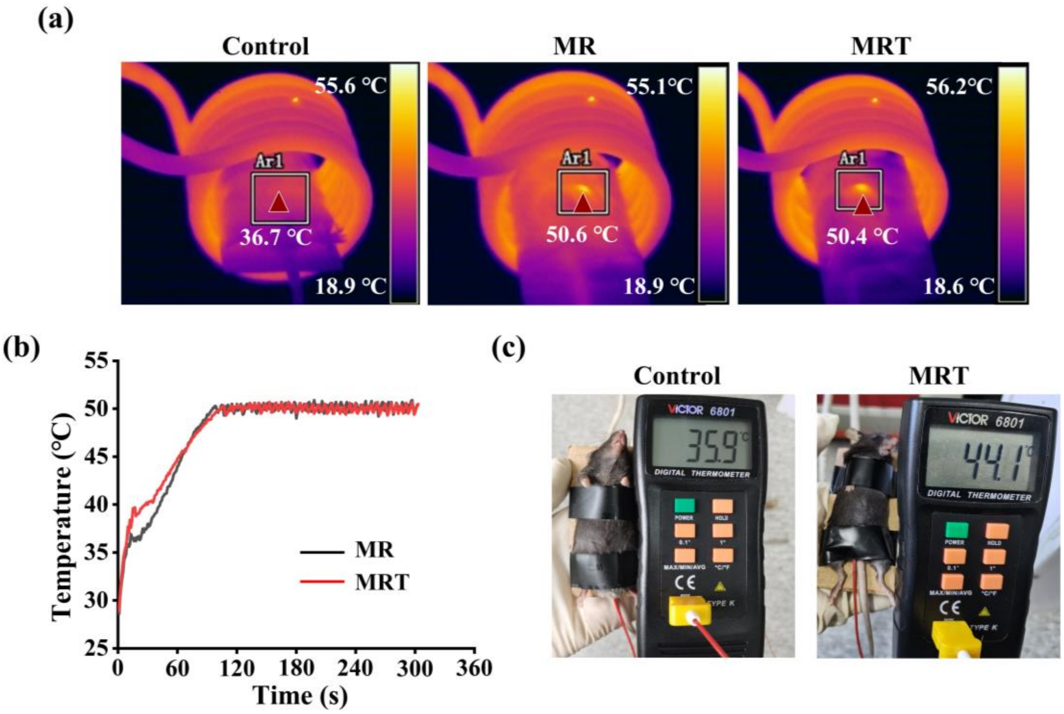

Figure S15. (a) Real-time *in vivo* IR thermal imaging of mice under AMF (1.7 mT) for 5 min when MR/MRT nanoparticles (6 mg/mL) were injected subcutaneously in the abdomen of mice. (b) The corresponding temperature-rising curves of MR/MRT group under AMF (1.7 mT) for 5 min. (c) The measured temperature in the designated site of colorectal region by thermocouple probe thermometer immediately after turning off AMF (the temperature loss during this period was ~6.5 ℃), the actual temperature of colorectal region in mice treated by MRT+MH was calculated according to the following formula: *Actual temperature* = *Measured temperature* + *Lost temperature* (n = 4).

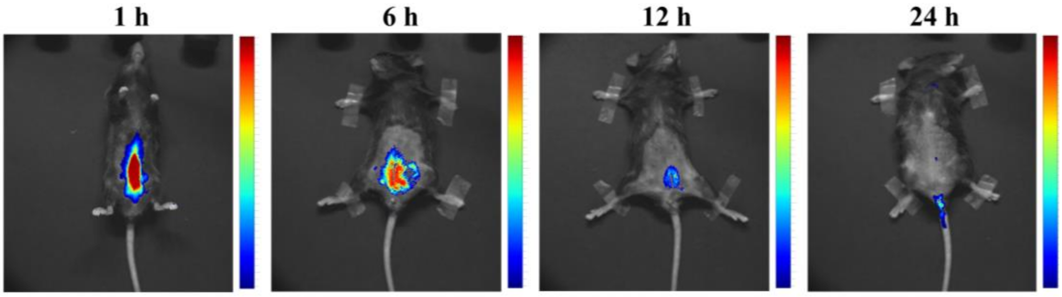

Figure S16. *In vivo* bioluminescence images of MRT nanoparticles (IR783) in the intestine of CRC model over time (1, 6, 12 and 24 h) after perfusion (n = 4).

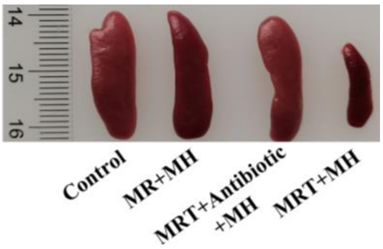

Figure S17. Representative digital photos of dissected spleens in various groups at the end of treatments (n = 4).

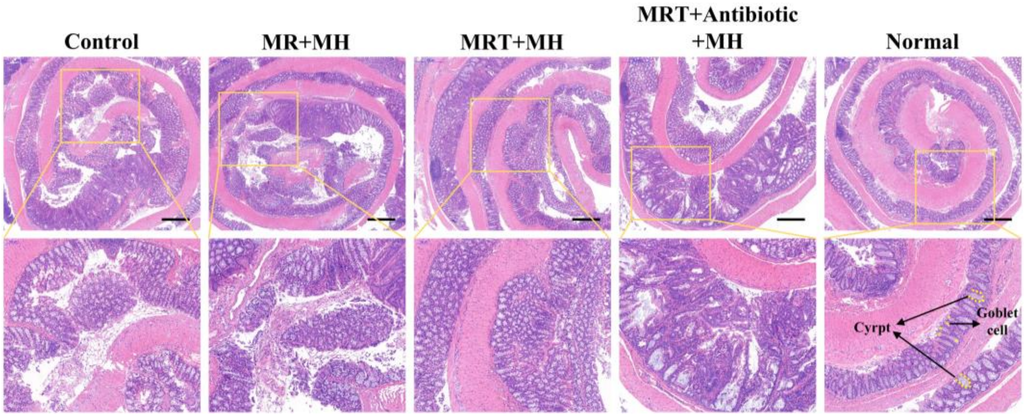

Figure S18. The H&E-stained distal colons of CRC mice in different groups at the end of treatments. Scale bar, 500 μm.

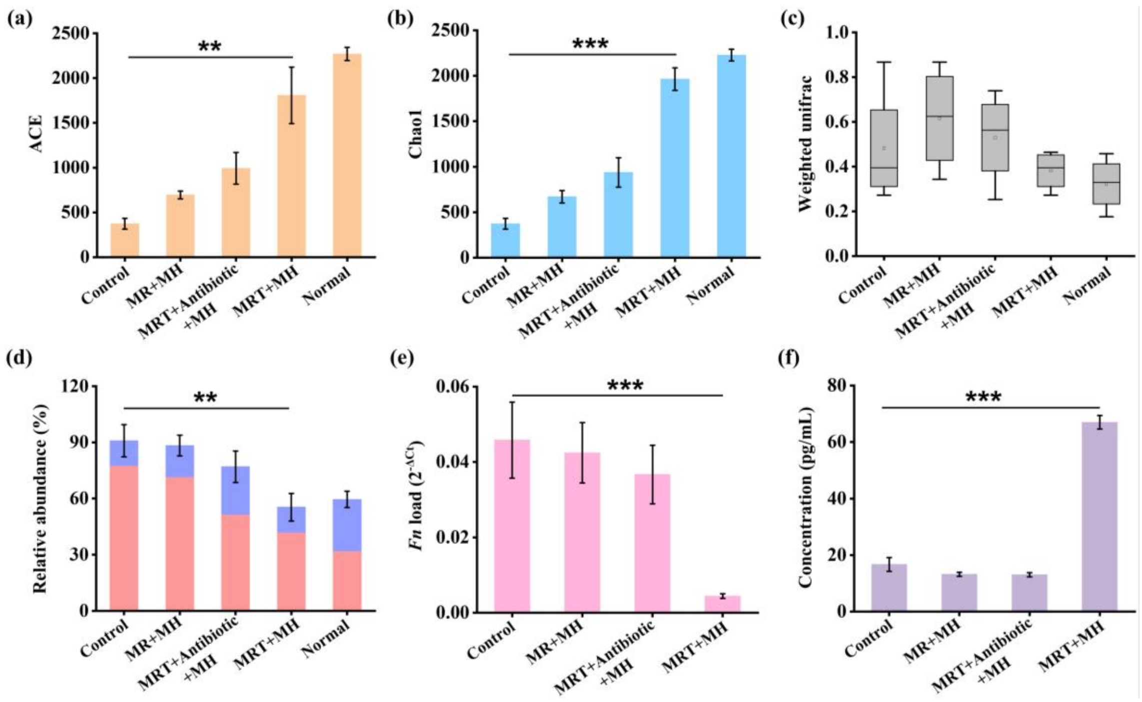

Figure S19. (a) The ACE index and (b) chao1 diversity indexes of intestinal bacteria in CRC mice determined by 16S rRNA high-throughput sequencing (n = 3). (c) The β diversity of intestinal bacteria in CRC mice was evaluated by weighted unifrac analysis according to the 16S rRNA results (n = 3). (d) The relative abundance of *Bacteroidota* (below) and *Firmicutes* (upper) in CRC mice evaluated by 16S rRNA analysis (n = 3). (e) Assessment of *Fn* load in the colorectal tissues by *Fn*-targeted qPCR (n = 3). (f) The LPS concentrations in the serum isolated from CRC mice (n = 3). Data are expressed as means ± SD (n = 3). Statistical significances were calculated via one-way ANOVA, *p < 0.05, **p < 0.01 and ***p < 0.001.

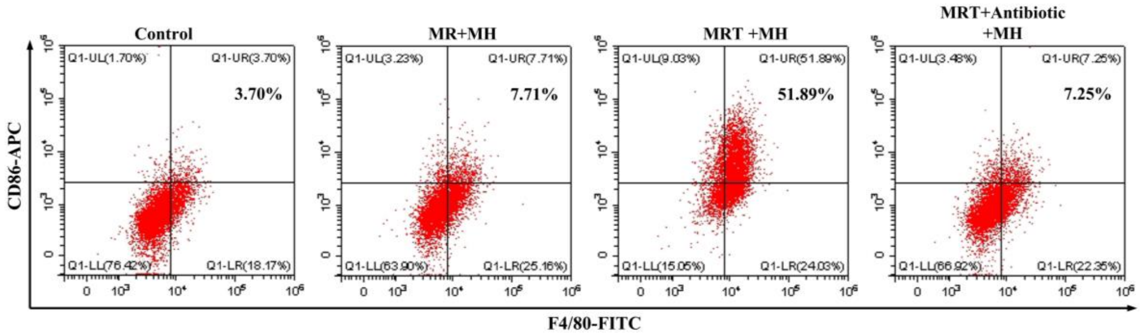

Figure S20. Representative flow cytometry plots indicate the percentages of $CD86^{+}F4/80^{+}$ macrophages (M1) in the harvested tumors on the 10th day after the first treatment (n = 3).

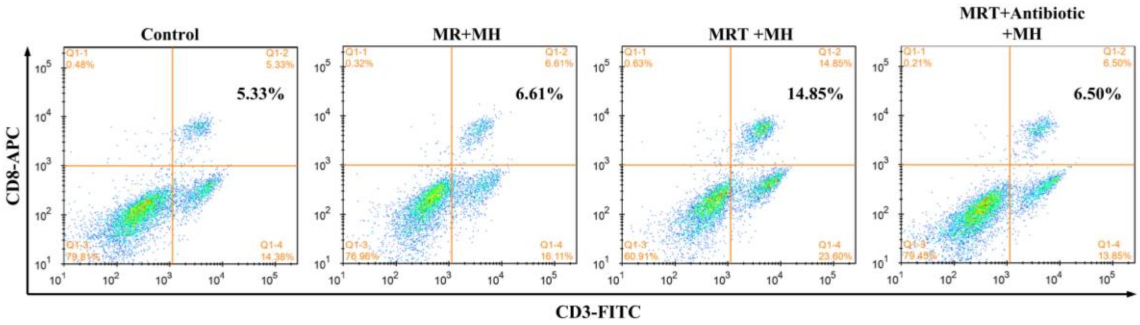

Figure S21. Representative flow cytometry plots indicate the proportions of $CD8^+$ cytotoxic T cells ($CD8^+CD3^+$) in the harvested spleen on the 10th day after the first treatment (n = 3).

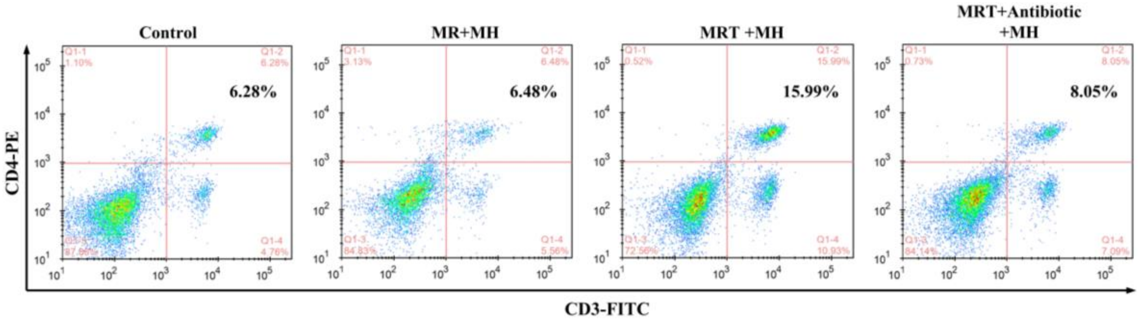

Figure S22. Representative flow cytometry plots indicate the proportions of $CD4^+$ T helper cells ($CD4^+CD3^+$) in the harvested spleen on the 10th day after the first treatment (n = 3).

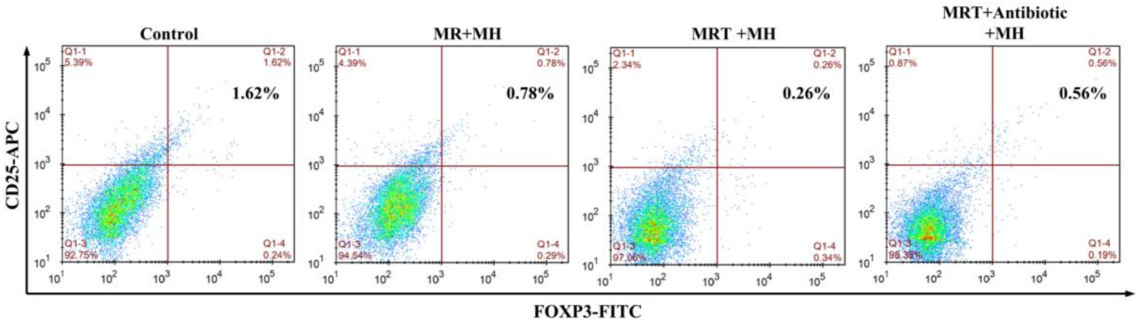

Figure S23. Representative flow cytometry plots indicate the proportions of $CD25^+Foxp3^+$ cells gated on $CD4^+$ T cells (Treg cells) in the harvested spleen on the 10th day after the first treatment (n = 3).

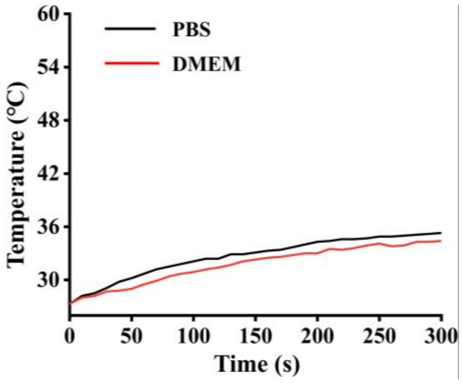

Figure S24. Temperature-time curves of PBS and DMEM solutions under AMF (1.35 kAm^{-1}).

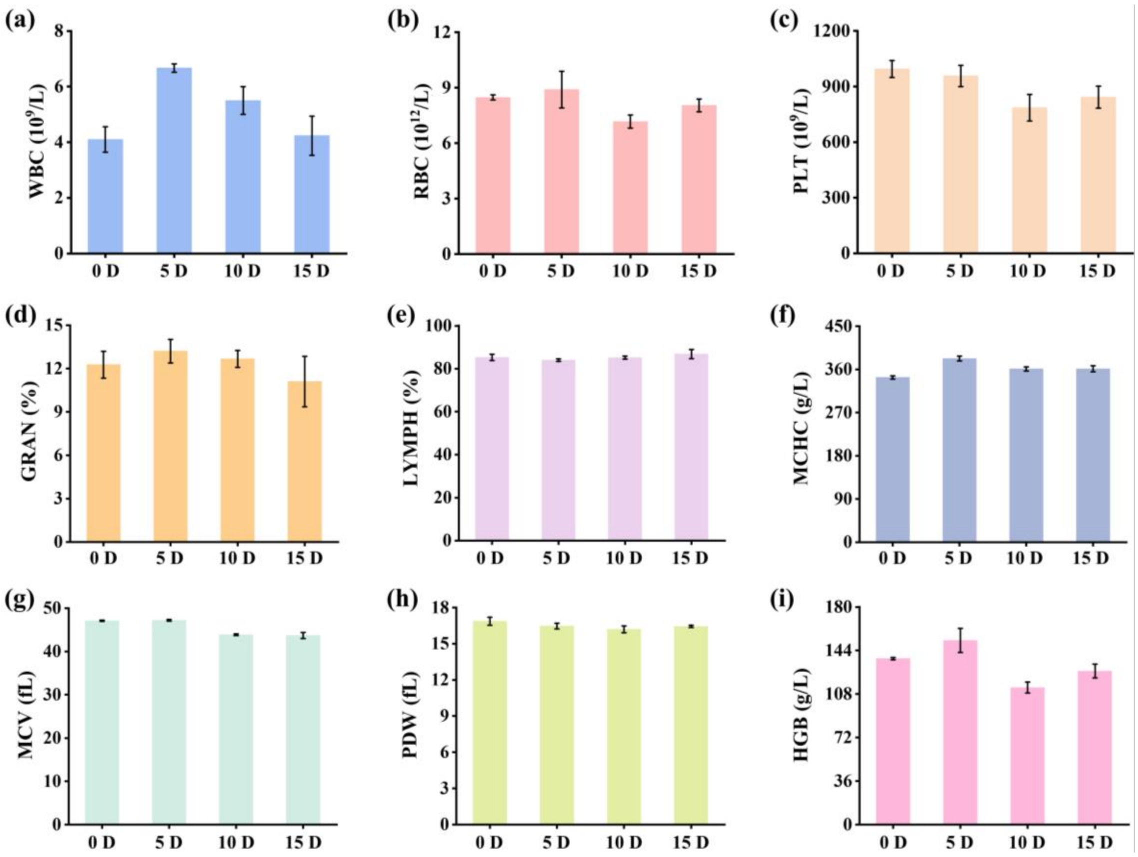

Figure S25. Complete blood panel assays of the blood samples after MRT+MH treatment for varied periods (0 D as control). (a) WBC; (b) RBC; (c) PLT; (d) GRAN%; (e) LYMPH%; (f) MCHC; (g) MCV; (h) PDW; (i) HGB. Data are expressed as mean standard ± errors (n = 3).

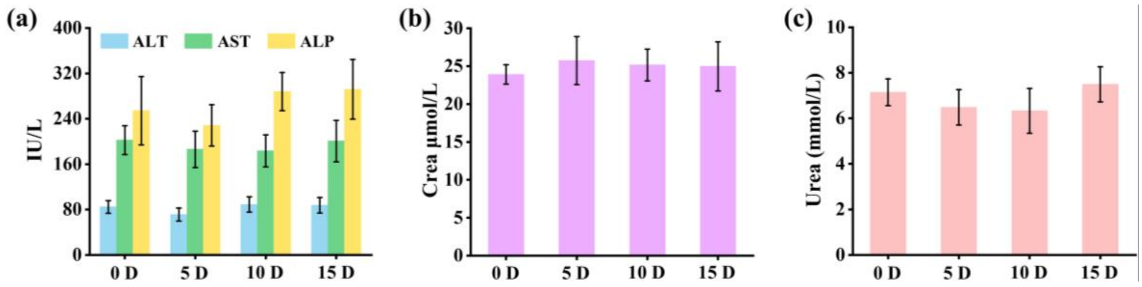

Figure S26. Blood chemistry profile analyses of the blood samples after MRT+MH treatments for varied periods (0 D as control). (a) ALT, AST and ALP; (b) Crea; (c) Urea. Data are expressed as mean standard ± errors (n = 3).

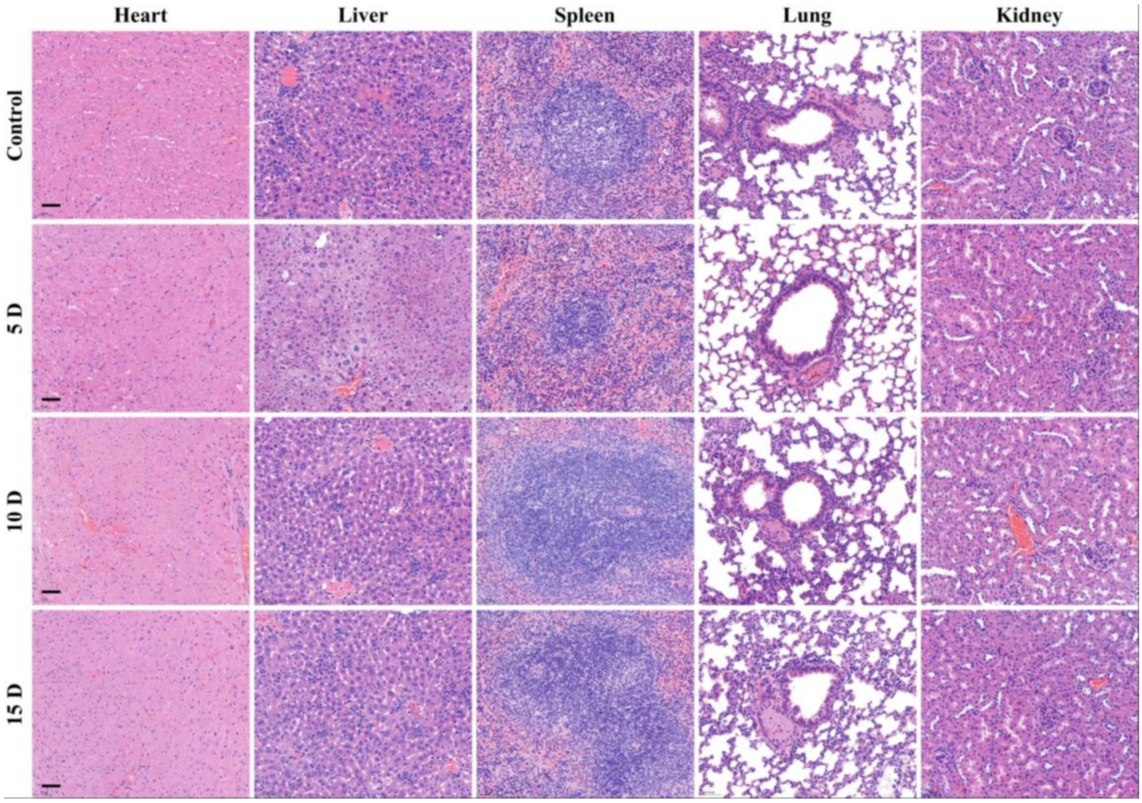

Figure S27. H&E staining images of the major organs (heart, liver, spleen, lung and kidney) of the mice after MRT+MH treatment for varied periods (0, 5, 10, 15 days, 0 D as control). Scale bar, 50 μm.

2. Supplementary Tables

Table S1. List of primers used in 16S rRNA sequencing.

**Gene (V3-V4)**	**Sequence (5'-3')**
**341F**	CCTAYGGGRBGCASCAG
**806R**	GGACTACHVGGGTWTCTAAT

Table S2. List of primers and Taqman probes used in qPCR.

**Gene**	**Sequence (5'-3')**	
**GAPDH**	Forward	GCACAGTCAAGGCCGAGAA
	Reverse	CCTCACCCCATTTGATGTTAGTG
	Probe	VIC-CATCACCATCTTCCAGGAGCGAGACC-BHQ1
Fusobacterium nucleatum	Forward	AAGCGCGTCTAGGTGGTTATGT
	Reverse	TGTAGTTCCGCTTACCTCTCCAG
	Probe	FAM-CAACGCAATACAGAGTTGAGCCCTGCATT-MGB

3. Supplementary Methods

Chemicals and reagents. Iron(III) acetylacetonate ($Fe(acac)_3$, 98%), cobalt(II) acetylacetonate ($Co(acac)_2$, 97%), oleylamine (80-90%), Iron(II) acetylacetonate ($Fe(acac)_2$, 98%, Meryer), N-hydroxysulfosuccinimide sodium salt (NHS, 98.0%), 1-ethyl-3-(3-dimethylaminopropyl)carbodiimide (EDC, 98.0%) and Dextran Sulfate Sodium Salt (DSS, 97%) were purchased from Aladdin Reagent (Shanghai) Co., Ltd.. Benzyl ether (95%) was purchased from Shanghai Macklin Biochemical Co., Ltd.. Oleic acid (≥90%), Bis(2,4-pentanedionato) manganese(II) dihydrate (≥98%), dimethyl sulfoxide (DMSO, 99%), zinc(II) acetylacetonate ($Zn(acac)_2$, ≥99%), (3-Carboxypropyl)Triphenylphosphonium bromide (TPP, ≥98%) and Methylene Blue Trihydrate (MB, 7220-79-3) were obtained from Adamas Reagent Co., Ltd.. 1,2-hexadecanediol (98%) was purchased from J&K Scientific Ltd.. Toluene (99.5%), hexamethylene (99.5%) and ethanol (99.7%) were bought from Shanghai Lingfeng Chemical Reagent Co., Ltd.. Arginyl-glycyl-aspartic acid (RGD, 98.0%) was provided by Shanghai BioChemPartner Co., Ltd.. 5,5-dimethyl-1-pyrroline N-oxide (DMPO, 99%) was acquired from Dojindo China Co., Ltd.. Azoxymethane (AOM, 25843-45-2) was bought from MPbio. NO assay kit (S0021S), BCA protein assay kit (P0012S) and RIPA lysis buffer (P0013B) was provided by Beyotime Biotechnology Co., Ltd.. Tachypleus Amebocyte Lysate (TAL, T125) was purchased from Charles River Laboratories International, Inc. Roswell park memorial institute 1640 (RPMI 1640), dulbecco’s modified eagle’s medium (DMEM), fetal bovine serum (FBS), antibiotics (10 000 U/mL penicillin and 10 000 μg/mL streptomycin), and 0.25% trypsin-EDTA solution were purchased from Gibco. Fluorescein isothiocyanate isomer I (FITC, 90%) was bought from BioFroxx. Collagenase IV (MX1004), Hyaluronidase (MX1007) and Deoxyribonuclease I (MF0402) were purchased

from Shanghai Maokang Biotechnology Co., Ltd.. Mouse TNF-α, Mouse IL-12 p70, Mouse IL-6, Mouse IL-1β ELISA Kit and Microbial lipopolysaccharide/endotoxin ELISA kit were purchased from Enzyme Linked (Shanghai) Biotechnology Co., Ltd.. Cell Counting Kit-8 (CCK-8), phosphate buffered saline solution (PBS, pH = 7.4), Simulated body fluid (SBF, pH = 7.4/6.5/5.5/4.0), Lipopolysaccharide (LPS) and HBSS/Ca/Mg (Gibco) were obtained from Shanghai Runcheng Biomedical Co., Ltd.. All of above chemicals were analytical grade and used as received without any further purification.

Synthesis of Zn^{2+}-doped $ZnCoFe_2O_4$@$ZnMnFe_2O_4$ magnetic nanoparticles (MNP). Oleic acid-coated MNP were synthesized by a previously reported method (1). In brief, $ZnCoFe_2O_4$ cores were synthesized as follows: $Fe(acac)_3$ (0.5 mmol), $Zn(acac)_2$ (0.1 mmol), $Co(acac)_2$ (0.15 mmol) and 1,2-hexadecanediol (2.5 mmol) were placed in 15 mL of benzyl ether, containing oleic acid (1.5 mmol) and oleylamine (1.5 mmol). The mixed solution was magnetically stirred and heated to 110 ℃ for 15 min in argon atmosphere, then refluxed (200 ℃) for 2 h. Afterwards, the mixture was heated to 290 ℃ and refluxed for 1 h under continuous stirring and argon flow. Then, the acquired solution containing $ZnCoFe_2O_4$ nanoparticles was cooled to room temperature and precipitated by adding 40 mL ethanol with further centrifugation (10000 rpm, 10 min). The obtained precipitate was dissolved in toluene (20 mL), containing oleic acid and oleylamine, and further centrifuged (6000 rpm, 10 min) to acquire the supernatant. The $ZnCoFe_2O_4$ was washed by ethanol and centrifuged (10000 rpm, 5 min) to remove the solvent. Finally, the $ZnCoFe_2O_4$ was redispersed in hexamethylene.

For the synthesis of $ZnCoFe_2O_4$@$ZnMnFe_2O_4$ nanoparticles, $ZnCoFe_2O_4$ nanoparticles were used as seeds and $ZnMnFe_2O_4$ were over-grown by thermal decomposition onto the surface of

the seed particles. $Fe(acac)_3$ (0.5 mmol), $Zn(acac)_2$ (0.1 mmol), $Mn(acac)_2$ (0.15 mmol), 1,2-hexadecanediol (2.5 mmol) and $ZnCoFe_2O_4$ were mixed in 15 mL of benzyl ether, containing oleic acid (1.5 mmol) and oleylamine (1.5 mmol). The following procedures were the same as the above method to obtain $ZnCoFe_2O_4$@$ZnMnFe_2O_4$ nanoparticles, but noteworthy is that the heating at 200 ℃ and 290 ℃ was reduced to 1 h and 30 min, respectively. Then $ZnCoFe_2O_4$@$ZnMnFe_2O_4$ nanoparticles with larger size were synthesized by further seed-mediated growth for five times. In each step, $ZnCoFe_2O_4$@$ZnMnFe_2O_4$ in 15 mL of benzyl ether were mixed with $Fe(acac)_3$ (0.5 mmol), $Zn(acac)_2$ (0.1 mmol), $Mn(acac)_2$ (0.15 mmol), 1,2-hexadecanediol (2.5 mmol), oleic acid (1.5 mmol) and oleylamine (1.5 mmol), the heating at 110 ℃, 200 ℃, and 290 ℃ was kept for 30 min, 1 h, and 30 min, respectively.

Modification of MNP nanoparticles. For dispersing in aqueous phase, toluene (5 mL) containing MNP (9 mg) was mixed with of deionized water (5 mL) containing RGD (30 mg) and then the mixture was stirred vigorously in a closed container. After 24 h, MNP modified by RGD (MR) could stably disperse in aqueous phase and the upper toluene was removed. The TPP complex was grafted onto MR nanoparticles by an EDC/NHS coupling reaction between $-NH_2$ on the surface of MR nanoparticles and -COOH on TPP. Briefly, DMSO solution of TPP complex (0.6 mmol) was added in deionized water containing EDC (0.4 mmol) and NHS (0.2 mmol). The obtained mixture was then stirred at room temperature for 30 min. Afterwards, MR in deionized water (1 mg/mL, 6 mL) was added in the mixture and continued stirring for 24 h. Excess EDC, NHS, and TPP was removed by repeatedly washing the obtained nanoparticles with deionized water for 3 times. Finally, the resultant MRT

nanoparticles were dispersed in deionized water.

Characterization of various nanoparticles. Transmission electron microscopy (TEM) images were acquired by using a field emission transmission electron microscope (TEM, JEOL JEM-2100, Japan). Energy-dispersive X-ray spectrometer (EDS) was measured using a spherical aberration-corrected field emission transmission electron microscope (ACTEM, Hitachi HF5000, Japan). Hydrodynamic sizes and zeta potentials were conducted on a Zetasizer system (Nano ZS90, Malvern Instrument Ltd.). Concentrations of metal elements in samples were analyzed by an inductively coupled plasma optical emission spectrometry (ICP-OES) (Agilent 725, Agilent Technologies, USA). The vibration of skeleton and infrared spectrum of organic function group in the samples were detected by Fourier Transform Infrared (FT-IR) Spectrometer (Nicolet iS10, recording range: 400-4000 cm^{-1}, the resolution: 4 cm^{-1}). The UV-Vis absorption spectra of various solutions were determined on UV-3600 spectrometer (Shimadzu). The field-dependent magnetization was measured using a Physical Property Measurement System (PPMS-9, Quantum Design, USA) at 300 K. The confocal fluorescence imaging was conducted on an FV1000 confocal microscope (Olympus Company, Japan). Flow cytometric analysis was performed on a flow cytometer (CytoFLEX, Beckman Coulter, USA).

Magnetic hyperthermia treatment of MR or MRT. The magnetic heating process of MR or MRT nanoparticles was conducted by a high-frequency induction heating equipment (SPG400K2). The alternating frequency and induction power were 577 kHz and 3.7 kW respectively. For magnetic hyperthermia (MH), nanoparticles at different concentrations were dispersed in 0.2 mL of PBS/SBF in a 2 mL microtube and the microtube was put inside the

copper coil. According to previous reports (2, 3), the temperature of physiological buffers would not be up-regulated under the alternating magnetic field (AMF) of 1.35 kAm^{-1} (B = 1.7 mT). Therefore, we recorded temperature of the solution by a thermal imager (Fotric 226s, China) during exposure to the AMF at 1.7 mT for 5 min.

Analysis of ·OH yield. The generation of hydroxyl radical (·OH) was detected by electron spin-resonance (ESR) spectroscopy. Briefly, the supernatants of MRT nanoparticles dispersed in SBF buffer solution (pH = 7.4, 6.5, 5.5, or 4.0, [Fe] = 5 mM for the nanoparticles) after MH treatment were collected and then mixed with H_2O_2 (400 μM). After 10 s of reaction, DMPO was added into the system and an aliquot of the mixture was extracted for ESR measurement. As for the quantitative analysis of ·OH generation, the supernatants of MRT nanoparticles dispersed in SBF at varied pH after MH treatment were collected, followed by the supplementation with H_2O_2 (5 mM) and MB (500 μg/mL). The variations of the absorbances at 664 nm of the mixtures were monitored using a UV-vis spectrometer.

Analysis of the stability of Fe element in MRT. The release of Fe in MRT was investigated in SBF (pH = 6.5 or 7.4) solution by ICP-MS at different time points (0/2/4/6/12/18/24/36/42/48/52 h) to evaluate the stability of MRT without MH treatment in simulated tumor microenvironment. MRT solution (2.5 mg/mL) was put into a dialysis bag (molecular weight: 8000) and then shaken slightly in a shaking table (37 ℃, 100 rpm). After certain time intervals, 6 mL solution of each was taken out to detect the Fe release amount by ICP-MS respectively, including 11 groups (0/2/4/6/12/18/24/36/42/48/52 h).

Cell culture

Preparation of BMDCs, BMDMs. BMDCs were generated as described previously (4). In

detail, after removing all muscle tissues with gauze from the femurs and tibias of C57BL/6 mice, the bones were placed in a 60 mm dish with 75% alcohol for 1 min, washed twice with PBS, and transferred into a fresh dish with RPMI 1640. Both ends of the bones were cut with scissors in the dish, and then the marrow was flushed out using 1 ml of RPMI 1640 with sterile syringe (1ml). The obtained tissue was suspended, passed through cell strainer (Falcon, 70 μm) to remove small pieces of bone and debris, and red cells were lysed with ACK lysis buffer. 1×10^6 ml^{-1} BM cells were cultured in RPMI 1640 containing 10% (vol/vol) FBS, 1% penicillin/streptomycin and 20 ng ml^{-1} GM-CSF. Cells were cultured at 37 ℃, and fresh medium was added every 2 or 3 days. Six days after the culture, the cells were determined by FACS detection of CD86, CD80 and MHC class II (MHC II) antigen, and collected for further experiments. The preparation of BMDMs was similar to the above. Briefly, BM cells after removal of red blood cells were cultured at 2×10^6 cells per well in six-well plates or 2×10^7 cells per 10 cm dish in DMEM containing 10% (vol/vol) FBS, 1% penicillin/streptomycin and 30% L929 conditioned medium. Fresh medium was added every 2 days. On day 6, BMDMs were collected for further experiments.

Culture of other cells. Mouse colon cancer cell line CT26 and mouse epithelioid fibroblasts cell line L929 were kindly provided by Cell Bank/Stem Cell Bank, Chinese Academy of Sciences. These cell lines were cultured in 75 cm^2 cell culture flasks containing RPMI 1640, with the addition of 10% FBS and 1% penicillin/streptomycin at 37 ℃ with 5% CO_2.

Culture of bacterial strains. *Fusobacterium nucleatum* (*Fn*) (ATCC 25586) was obtained from the American Type Culture Collection (ATCC). *Bifidobacterium pseudolongum* (*Bp*) (JCM1205) was obtained from Japan Collection of Microorganisms (JCM). *Escherichia coli* (*Ec*) and *Staphylococcus aureus* (*Sa*) were kindly provided by Shanghai Jiao Tong University

Affiliated Sixth People's Hospital. Brain heart infusion (BHI) broth, man rogosa sharpe (MRS) media and tryptic soy broth (TSB) media broth were purchased from Beina Biotechnology and used for the bacteria culture, respectively. All bacteria were purified from colonies isolated from plates for subsequent liquid subcultures according to the manufacturer's protocol.

***In vitro* antibiosis assay by spread plate method (SPM).** Suspensions of above four bacteria were anaerobically incubated in six-well microplates to reach the logarithmic phase (OD_{600} between 0.5 and 0.7) and diluted with suitable media to 10^7 colony-forming units (CFU/mL) followed by the addition of MR/MRT nanoparticles (400 μg/mL) for desired time (1-2 h) at 37 ℃. Afterwards, these bacteria were centrifuged and resuspended in a sterile microtube with SBF (pH = 6.5, 0.2 mL) containing H_2O_2 (100 μM), which was simulated as the tumor microenvironment, and then placed in the copper coil for MH treatment. Correspondingly, the bacteria without the addition of MR/MRT nanoparticles was regarded as control group. After MH treatment, these bacteria were collected and ten-fold diluted into a series of gradients (10^{-1}~10^{-7}) with PBS. Subsequently, 100 μL of these dilutions were extracted and spread onto columbia blood agar plates (CBA). Following overnight incubation at 37 ℃, we chose the CBA plates with uniform and countable bacterial colonies under the same dilution (10^{-4}), and the antibacterial effects of various groups were evaluated by the $Log_{10}(C)$ according to the following equation: $C = N \times D$, where C is the concentrations of various bacteria, N is the number of bacterial colonies and D is the initial dilution ratio. The experimental procedure described above is called the spread plate method (SPM).

On the other hand, we also detected the OD_{600} of bacteria treated by MRT at different

concentrations (0/50/100/200/400 μg/mL) with or without MH.

Bio-TEM observation. *Fn* strain was co-cultured with MR/MRT nanoparticles (400 μg/mL) for 2 h with or without MH. Then, the bacteria were collected and washed with PBS by centrifuging at 1000 r/min for 2-3 times. After the removal of supernatants, the bacteria were fixed by glutaraldehyde (2.5%) for 24 h at 4 ℃ and rinsed with PBS followed by being dehydrated through a graded ethanol series (30%, 50%, 70%, 80%, 90%, 95% and 100%) for 20 min at room temperature for each gradient, then 100% acetone twice. Subsequently, the bacteria sample was embedded in EPOM812 and then polymerized in the baking oven at 37 ℃, 45 ℃ and 60 ℃ for 12 h, 12 h and 48 h, respectively. Ultrathin sections of 60-80 nm thick were cut with a diamond knife on a Leica UC6 ultramicrotome and transferred to the copper grid.

***Limulus* amebocyte lysate assay.** Tachypleus amebocyte lysate (0.25 EU/mL) was used to qualitatively detect endotoxin (LPS) in the supernatants of *Fn* (10^5 CFU/mL) treated by MR/MRT with or without MH according to the manufacturer's protocol. In brief, we used DMEM medium to dissolve the *limulus* lysate. Then we added bacterial supernatants of different groups into the test tubes containing the *limulus* lysate and placed them in the incubator at 37 ℃ for 1 h. Finally, we observed whether there was a stable gel formation in each test tube. Additionally, the concentrations of LPS released by *Fn* after various treatments were also determined using an ELISA kit according to the manufacturer's instructions.

Preparation of conditioned medium containing *Fn* supernatant. *Fn* (10^9 CFU/mL) were anaerobically cocultured with MR/MRT nanoparticles (400 μg/mL) for 2 h. After MH treatment, the supernatants of *Fn* were collected by magnetic separation and centrifugation.

Subsequently, we added BenzoNuclease (GMP grade) to remove the bacterial nucleic acids and then boiled the supernatants at 100 ℃ for 10 minutes to inactivate the residual proteins in the supernatants (the bioactivity of bacterial endotoxin lipopolysaccharide was hardly affected at 100 ℃). In order to remove the bacteria with intact biofilms remained in the supernatants, we further filtered the above supernatants through an injection filter (0.2 μm, PES). The obtained bacterial supernatants were diluted 1:20 with fresh DMEM or RPMI 1640 into conditioned media for subsequent experiments.

***In vitro Fn* after various treatments binding to CT26 assay.** *Fn* treated by MR/MRT with or without MH were labeled with green fluorescent FITC according to the reported method (5). In order to avoid the influence of various nanoparticles on CT26 cells, we used a strong magnetic magnet to separate the bacteria and nanoparticles before co-incubation. Then, various groups of FITC-labeled *Fn* (10^5 CFU/mL) were co-incubated with CT26 cells (1×10^4) in confocal dishes for 2 h and then washed for 2 times. For confocal laser scanning microscopic (CLSM) imaging, CT26 cells adhere at the bottom of confocal dishes were fixed with methanol. FITC fluorescence (green) of *Fn* and morphology of CT26 was observed by CLSM and the quantitative analysis of fluorescence intensity was performed with ImageJ software.

Western Bolt Analysis. In order to avoid the influence of various nanoparticles on CT26 cells, we used a strong magnetic magnet to separate the bacteria and nanoparticles before co-incubation. After co-incubation with *Fn* treated by MR/MRT (400 μg/mL) (with or without MH), the CT26 cells were digested with trypsin (0.25%, Sigma) and washed for 3 times. The lysates of CT26 cells were collected by RIPA extraction kit and concentration of protein was

detected by BCA protein assay kit according to the manufacturer's protocol. Various primary antibodies were co-incubated with polyvinylidene fluoride (PVDF) after wet transfer. The primary antibodies used were as follows: anti-HMGB1 (ab18256, Abcam), anti-TGF-β1 (ab215715, Abcam), anti-GAPDH (60004-1-Ig, PTG).

The BMDMs stimulated by the above conditioned media containing supernatants of *Fn* for 12 h were collected and the protein lysates of BMDMs were extracted according to the manufacturer's protocol, in which commercial LPS (10 μg/mL) was used as a positive control. The western blotting procedure was carried out by the manufacturer's protocol and primary antibodies were used as follows: anti-MyD88 (ab219413, abcam), anti-TLR4 (bs-2717R, Bioss Biotechnology), anti-NF-κB p65 (bs-0465R, Bioss Biotechnology), anti-GAPDH (60004-1-Ig, PTG).

Cell viability assay. For evaluating the cytotoxicity of macrophages against tumor cells, BMDMs were stimulated with conditioned media containing supernatants of *Fn* after various treatments and then cocultured with CT26 cells for 24 hours. The viability of cocultured cells was investigated by the standard cell-counting kit 8 (CCK-8) following the manufacturer's protocol. Firstly, BMDMs were seeded in a 96-well plate overnight (initial density: 1×10^4 cells per well), then treated with conditioned media containing supernatants of *Fn* for 12 h, in which commercial LPS was used as a positive control. After that, CT26 cells (4×10^3 cells per well) were added to above 96-well plate and cocultured with BMDMs for 24 h. It is necessary to set up two blank groups in which BMDMs and CT26 cells were cultured separately without any treatment. Finally, the CCK-8 kit was applied to test cell viabilities in each experiment group. The killing efficiency of BMDMs on CT26 cells was calculated according to the

following formula: $K = [1 - (M - B)/C] \times 100\%$, where M indicates the viability of cocultured BMDMs and CT26 in control/MR+MH/MRT+MH/LPS group, B indicates the viability of BMDMs without any treatment, and C indicates the viability of CT26 without any treatment.

Flow cytometry

Analysis of M1 phenotype in vitro**.** BMDMs were incubated in 6-well plates (2×10^6 per well) as described in the cell culture section. After the addition of various stimulation (conditioned media containing supernatants of *Fn* treated by MR+MH/MRT+MH), the macrophages were collected by centrifugation and washed for 2-3 times, followed by co-incubation with anti-CD86 APC (105011, BioLegend) and anti-F4/80 FITC (123107, BioLegend) at 4 ℃ according to the manufacturer's protocol. Then the cells were gently rinsed with PBS containing 0.1% BSA three times and analyzed for the M1 phenotype of BMDMs using a flow cytometer (CytoFLEX, Beckman Coulter). The data were collected via the software CytExpert (version 2.2) and then analyzed by FlowJ (version 10.0).

On the other hand, before the above various stimulation of BMDMs, we added IL-4 cytokine (10 ng/mL) to induce the M2 transformation of BMDMs in the process of culture. Subsequently, we stimulated M2 macrophages by conditioned media containing supernatants of *Fn* and detected the M2 to M1 subtype transformation of BMDMs by flow cytometry, in which the cells were stained with anti-CD206 PE (141705, BioLegend) and anti-F4/80 FITC (123107, BioLegend) under the manufacturer's protocol.

Stimulation of BMDCs in vitro**.** For BMDCs stimulation *in vitro*, BMDCs were incubated with the conditioned media containing supernatants of *Fn*, commercial LPS (10 μg/mL) was used as a positive control. After 12 h, BMDCs were collected and stained with anti-CD80 PE

(104707, Biolegend), anti-CD86 FITC (105005, Biolegend), anti-CD86 PE (159203, Biolegend) and anti-MHC II FITC (109905, Biolegend) under the manufacturer's protocol. Finally, flow cytometry (CytoFLEX, Beckman Coulter) was used for analyzing the maturation and antigen presentation of BMDCs.

ELISA. *In vitro* the immune-associated cytokines (i.e., IL-6, IL-1β, TNF-α and IL-12 p70) secreted into the supernatants of BMDMs were quantified by using commercial ELISA kits with a standard protocol. In the same way, serum samples were isolated from mice on the 10th day after the first treatment for analysis *in vivo* according to vendors' protocols. Additionally, the concentration of LPS released by *Fn* after various treatments in the serum were also determined using an ELISA kit according to the manufacturer's instructions.

***In vivo* anticancer efficacy evaluation of MRT+MH**

The establishment of CRC model. All animal experiment procedures follow the guidelines of the Animal Care Ethics Commission of Shanghai Tenth People's Hospital, Tongji University School of Medicine (ID: SHDSYY-2018-Z0026). Sixty healthy male C57BL/6 mice (4 weeks, SPF) were purchased from Beijing Vital River Laboratory Animal Technology Co., Ltd. These mice were housed in ventilated and sterilized cages under standard conditions (light: 12 h light/dark cycle, ambient temperature: 25 ± 2 ℃, humidity: 60 ± 10%, ultraviolet germicidal lamp irradiation about 40 min daily), which were fed with pellet food ad libitum and sterilized water. Then, the mice were treated with AOM (10 mg/kg) via intraperitoneal injection; after 5 days, the mice were fed with 2 wt/vol % DSS in drinking water for 5 days. Subsequently, they were fed with normal drinking water for 15 days. The drinking water containing DSS (2 wt/vol %) and normal drinking water are alternately replaced each other for three times. The

occurrence of intestinal tumors was detected and analyzed by anatomical observation and H&E-stained colon sections on the 83th day. The length and width of tumor in CRC mice were measured and the tumor volumes were calculated according to the following equation: $(\text{width}^2 \times \text{length})/2$.

The treatment of CRC model. On the seventh day after successful establishment of CRC model, named as day 0, forty mice were randomly selected from the above CRC model mice and divided into four groups as follows (n = 10): (1) Control, (2) MR+MH, (3) MRT+MH, (4) MRT+Antibiotic+MH, in which a cocktail formula of antibiotics contained ampicillin (1 mg/mL), gentamicin (1 mg/mL), metronidazole (1 mg/mL), and vancomycin (0.5 mg/mL). Then, all CRC mice in each group were administered by PBS/MR/MRT/MRT+Antibiotics respectively by rectal perfusion with gastric lavage needle (MR/MRT: ~15 mg/kg), wherein mice in the fourth group were first perfused with MRT for 1 hours and then with antibiotics. After perfusion, the mice were gently placed in cages belly up for 30 min and then transferred to AMF (1.7 mT) for 5 min. Thermocouple probe thermometer (DW6801A, China) and far-infrared thermometer (Fotric 225, USA) were used to detect the temperature changes in colorectal region of mice. The perfusion administrations and MH treatments of four groups were conducted on day 0 and day 7, and they were stopped feeding 24 hours before and during the perfusion administrations. During the treatment, the weight of mice in all groups were measured once every two days for 18 days. At the end of treatment, experimental mice of different groups were euthanized at day 18, and the colorectal tissues were dissected to count the number of tumors, measure the size of tumors, photograph and further conduct H&E analysis.

In vivo analysis of TAMs and T cells. On the 10th day after the first treatments, the blood

samples, colon and spleen tissues were harvested and collected from random three mice in each group. The serum was obtained by centrifugation of the blood samples at 2000 g for 20 minutes at 4 ℃. Meanwhile, the tumors on colorectal tissues of each group were dissected, cleaned, cut into small pieces and digested using collagenase IV (175 U/ml, NEOFROXX), hyaluronidase (100 U/ml, NEOFROXX), and deoxyribonuclease (30 U/ml, NEOFROXX) at 37℃ for 60 min, then filtered through 70-μm cell strainer (Falcon) to acquire single-cell suspensions. The cells were incubated with anti-CD86 APC (105011, BioLegend) and anti-F4/80 FITC (123107, BioLegend) at 4 °C according to the manufacturer’s protocol. In addition, the single-cell suspensions of spleen tissues were also prepared by piston grinding with 5ml sterile syringe and filtration with cell strainer. The cells in spleen were incubated with anti-CD3 FITC (100203, BioLegend), anti-CD4 PE (100408, BioLegend), anti-CD8a APC (100712, BioLegend), anti-FOXP3 FITC (126405, BioLegend) and anti-CD25 APC (101909, BioLegend) at 4 °C according to the manufacturer’s protocol. Finally, flow cytometry was used for cell sorting and analyzing.

Bacterial 16S ribosomal RNA (rRNA) gene sequencing. Stool samples were freshly collected from above treated mice and healthy mice for intestinal bacterial diversity analysis on the 6th day after the first treatment (n = 3) as described previously (6). DNA was extracted from murine fecal samples using an isopropanol DNA precipitation method (https://www.qiagen.com/us/knowledge-and-support/knowledge-hub/bench-guide/dna/). The DNA was recovered with 30 ml of buffer in the kit. PCR products were mixed in equidensity ratios. Then, the mixture of PCR products was purified with Qiagen Gel Extraction Kit (28706, Qiagen, Germany). The bacterial 16S primers were used to amplify the *V3-V4* region

of the 16S rRNA gene. These primers have been custom-designed by Novogene Bioinformatics Technology to provide the best coverage of the 16S gene while maintaining high sensitivity. The amplification was performed at a higher annealing temperature to ensure only bacterial sequences were amplified. All PCR reactions were carried out with Phusion® High-Fidelity PCR Master Mix with GC Buffer (New England Biolabs). Sequencing libraries were generated using TruSeq® DNA PCR-Free Sample Preparation Kit (Illumina, USA) following manufacturer's recommendations and index codes were added. The library quality was assessed on the Qubit@ 2.0 Fluorometer (Thermo Scientific) and Agilent Bioanalyzer 2100 system. At last, the library was sequenced on NovaSeq6000 platform and 250 bp paired-end reads were generated. The acquired sequencing data were processed and analyzed using FLASH (V1.2.7, http://ccb.jhu.edu/software/FLASH/), QIIME (Version 1.9.1, http://qiime.org/scripts/split_libraries_fastq.html), Uparse (Uparse v7.0.1001 ， http://www.drive5.com/uparse/) and RDP classifier (Version 2.2, http://sourceforge.net/projects/rdpclassifier/) following the manufacturer’s instructions. The primers are listed in Supplementary Table S1.

Quantitative PCR (qPCR) of *Fn* in the colorectal tissue. A custom TaqMan primer/probe set was used to amplify *Fn* genus DNA (Integrated DNA technologies, CA) as previously described (7). The cycle threshold (Ct) values for *Fn* genus was normalized to the amounts of mouse genomic DNA in each reaction by using a primer and probe set for the endogenous reference gene GAPDH, and the fold difference ($2^{-\Delta Ct}$) of *Fn* load in the colorectal tissue was calculated as described before (8). Briefly, stool pellet, luminal content, and washed colorectal tissue samples were collected and then total DNA was extracted on the 6th day after the first

treatment using the TIANamp Stool DNA Kit (DP328) following the manufacturer's protocol. The quantitative real-time PCR (qPCR) was carried out using a reaction volume of 10 μL consisting of Taqman Universal qPCR master mix (Power SYBR-ABI), 10 μM of each primer and the respective probe (0.4 μL). The qPCR conditions were initially heating at 95 ℃ for 10 min to activate the Taq Polymerase, finally 40 cycles at 95 ℃ for 15 s and 60 ℃ for 1 min. The data was acquired at the end of each cycle. Both the primers and probes were purchased from Jieli Biotechnology (Shanghai) Co., Ltd.. The primer and probe sequences for qPCR are listed in Supplementary Table S2.

Immunohistochemistry staining. Distal colons were harvested from mice in different groups on the 10th day after the first treatment and fixed in 4% paraformaldehyde for immunohistochemistry staining according to the experimental instruction. The tissue sections were used for antigen repair and were put into 3% hydrogen peroxide solution to block endogenous peroxidase. Afterwards, the tissue sections were incubated with primary antibodies CD86 (DF6332, Affinity) and F4/80 (DF2789, Affinity) overnight at 4 ℃. Then, the sections were incubated with dye-conjugated secondary antibodies at room temperature for 1 h. After being counterstained with DAPI for another 10 min, the sections were then washed twice with PBS and observed by CLSM.

4. Supplemental References

1. Pan J, Xu Y and Wu Q *et al.* Mild magnetic hyperthermia-activated innate immunity for liver cancer therapy. *J Am Chem Soc* 2021; **143**: 8116-28.
2. Pan J, Hu P and Guo Y *et al.* Combined magnetic hyperthermia and immune therapy for primary and metastatic tumor treatments. *ACS Nano* 2020; **14**: 1033-44.
3. Wang J, Wang L and Pan J *et al.* Magneto-based synergetic therapy for implant-associated infections via biofilm disruption and innate immunity regulation. *Adv Sci* 2021; **8**: 2004010.
4. Yao Y, Chen S and Cao M *et al.* Antigen-specific CD8(+) T cell feedback activates NLRP3 inflammasome in antigen-presenting cells through perforin. *Nat Commun* 2017; **8**: 15402.
5. Dong X, Pan P and Zheng D-W *et al.* Bioinorganic hybrid bacteriophage for modulation of intestinal microbiota to remodel tumor-immune microenvironment against colorectal cancer. *Sci Adv* 2020; **6**: eaba1590.
6. Li Y, Dong J and Xiao H *et al.* Gut commensal derived-valeric acid protects against radiation injuries. *Gut Microbes* 2020; **11**: 789-806.
7. Boutaga K, Van Winkelhoff A-J and Vandenbroucke-Grauls C-M *et al.* Periodontal pathogens: a quantitative comparison of anaerobic culture and real-time PCR. *FEMS Immunol Med Microbiol* 2005; **45**: 191-9.
8. Castellarin M, Warren R-L and Freeman J-D *et al.* Fusobacterium nucleatum infection is prevalent in human colorectal carcinoma. *Genome Res* 2012; **22**: 299-306.
